# Analysis of the human Y-chromosome haplogroup Q characterizes ancient population movements in Eurasia and the Americas

**DOI:** 10.1186/s12915-018-0622-4

**Published:** 2019-01-24

**Authors:** Viola Grugni, Alessandro Raveane, Linda Ongaro, Vincenza Battaglia, Beniamino Trombetta, Giulia Colombo, Marco Rosario Capodiferro, Anna Olivieri, Alessandro Achilli, Ugo A. Perego, Jorge Motta, Maribel Tribaldos, Scott R. Woodward, Luca Ferretti, Fulvio Cruciani, Antonio Torroni, Ornella Semino

**Affiliations:** 1 Dipartimento di Biologia e Biotecnologie “L. Spallanzani”, Università di Pavia, Via Ferrata, 9, 27100 Pavia, Italy; 2 Estonian Biocentre, Institute of Genomics, University of Tartu, Tartu, Estonia; 3 Dipartimento di Biologia e Biotecnologie “C. Darwin”, Sapienza Università di Roma, Rome, Italy; 4 Secretaría Nacional de Ciencia, Tecnología e Innovación (SENACYT), Panama City, Panama; 5 Department of Health Technology Assessment and Economic Evaluation, Panama City, Panama; 6 Department of Biology, Utah Valley University, Orem, UT USA

**Keywords:** Human Y-chromosome variation, Haplogroup Q phylogeny, Origin of Native Americans, Origin of Eurasians, Peopling of the Americas

## Abstract

**Background:**

Recent genome studies of modern and ancient samples have proposed that Native Americans derive from a subset of the Eurasian gene pool carried to America by an ancestral Beringian population, from which two well-differentiated components originated and subsequently mixed in different proportion during their spread in the Americas. To assess the timing, places of origin and extent of admixture between these components, we performed an analysis of the Y-chromosome haplogroup Q, which is the only Pan-American haplogroup and accounts for virtually all Native American Y chromosomes in Mesoamerica and South America.

**Results:**

Our analyses of 1.5 Mb of 152 Y chromosomes, 34 re-sequenced in this work, support a “coastal and inland routes scenario” for the first entrance of modern humans in North America. We show a major phase of male population growth in the Americas after 15 thousand years ago (kya), followed by a period of constant population size from 8 to 3 kya, after which a secondary sign of growth was registered. The estimated dates of the first expansion in Mesoamerica and the Isthmo-Colombian Area, mainly revealed by haplogroup Q-Z780, suggest an entrance in South America prior to 15 kya. During the global constant population size phase, local South American hints of growth were registered by different Q-M848 sub-clades. These expansion events, which started during the Holocene with the improvement of climatic conditions, can be ascribed to multiple cultural changes rather than a steady population growth and a single cohesive culture diffusion as it occurred in Europe.

**Conclusions:**

We established and dated a detailed haplogroup Q phylogeny that provides new insights into the geographic distribution of its Eurasian and American branches in modern and ancient samples.

**Electronic supplementary material:**

The online version of this article (10.1186/s12915-018-0622-4) contains supplementary material, which is available to authorized users.

## Background

There is a general agreement that anatomically modern humans entered the American continent from Beringia between 20 and 15 thousand years ago (kya), and two possible routes, one coastal and one inland, have been postulated [1, 2]. The first route, accessible since 20 kya, would have probably facilitated a rapid southward expansion along Pacific coastal regions of the double continent, while the second one, through the so-called ice-free corridor between the Cordilleran and Laurentide ice sheets, might have been accessible from 15.6–14.8 kya [3] and, according to some models, would have contributed solely or mainly to the peopling of North America. The most favoured scenario by studies of modern and ancient nuclear data [4–6] is that both Athabascans and Amerindians derive from the same founding Beringian population, which entered America prior to 13 kya, and that the split between northern and southern Native Americans occurred south of the North American ice sheets. This was followed by two additional minor gene introgressions restricted to the Arctic region: the Saqqaq/Dorset Paleo-Eskimo ~ 4.5 kya and the Thule-related Neo-Eskimo ~ 2 kya.

Until recently, most of the genetic information concerning the first peopling of the Americas was largely derived from the maternally transmitted mitochondrial DNA (mtDNA), with only a few mtDNA haplogroups (Hgs) (A2, B2, C1, C4c, D1, D4h3a and X2a) [7–9], nested within Eurasian clades, characterizing almost all present Native Americans. According to mtDNA studies of the last decade, these haplogroups entered the Americas around 16 kya [10, 11], after a rather long period of standstill and differentiation in Beringia [12], possibly following two entry routes, the first along the Pacific coast marked by D4h3a and the second through the ice-free corridor marked by X2a and C4c [10, 13–16]. At the moment, the major difference between the conclusions of mtDNA and autosomal DNA studies appears to concern the location of the split between the ancestors of northern and southern Native Americans. Indeed, a split of the first settlers in eastern Beringia (Alaska) rather than south of the Cordilleran and Laurentide ice sheets would imply a dual rather than a single entry into the American continent. If so, the two entries could have occurred either at the same time or at different times, following the same or different routes [17].

Unfortunately, the identification of Native American founding lineages of the male-specific region of the Y-chromosome (MSY) has been complicated by the post-Columbian uneven male/female native population decline and by the high historical rate of male-mediated admixture into Native American communities. Nevertheless, two founding lineages of Asian origin, Hg C and Hg Q, were described long ago [18]. Hg C is virtually limited to North America while Hg Q-M242 is present as Q-L54 all over the double continent with two main Native American founding sub-lineages: Q-M3 and Q-L54*(xM3, L330) [19, 20]. Little information is available about the distribution of these two sub-lineages, except that they arrived concomitantly in Mesoamerica, where Mexico acted as a recipient for the first migration wave, followed by a rapid southward spread into the southern continent [20, 21]. In the last years, thanks to the advances in DNA sequencing technologies, which allow large-scale analyses of nearly complete Y-chromosome sequences, and the increasing participation of citizens to genealogical projects (International Society of Genetic Genealogy), new L54 sub-lineages have been identified [22–25]. Yet, both the current level of resolution of haplogroup Q and its phylogeography remain inadequate to explore the history and demography of Native American populations from a Y-chromosome perspective.

To provide new clues on the genetic history of the Americas, here, we present a comprehensive re-assessment of the Pan-American Y-chromosome haplogroup Q-L54, including a detailed reconstruction of its phylogeography and a description of its relationships with the Eurasian branches of haplogroup Q.

## Results

**Fig. 1:**
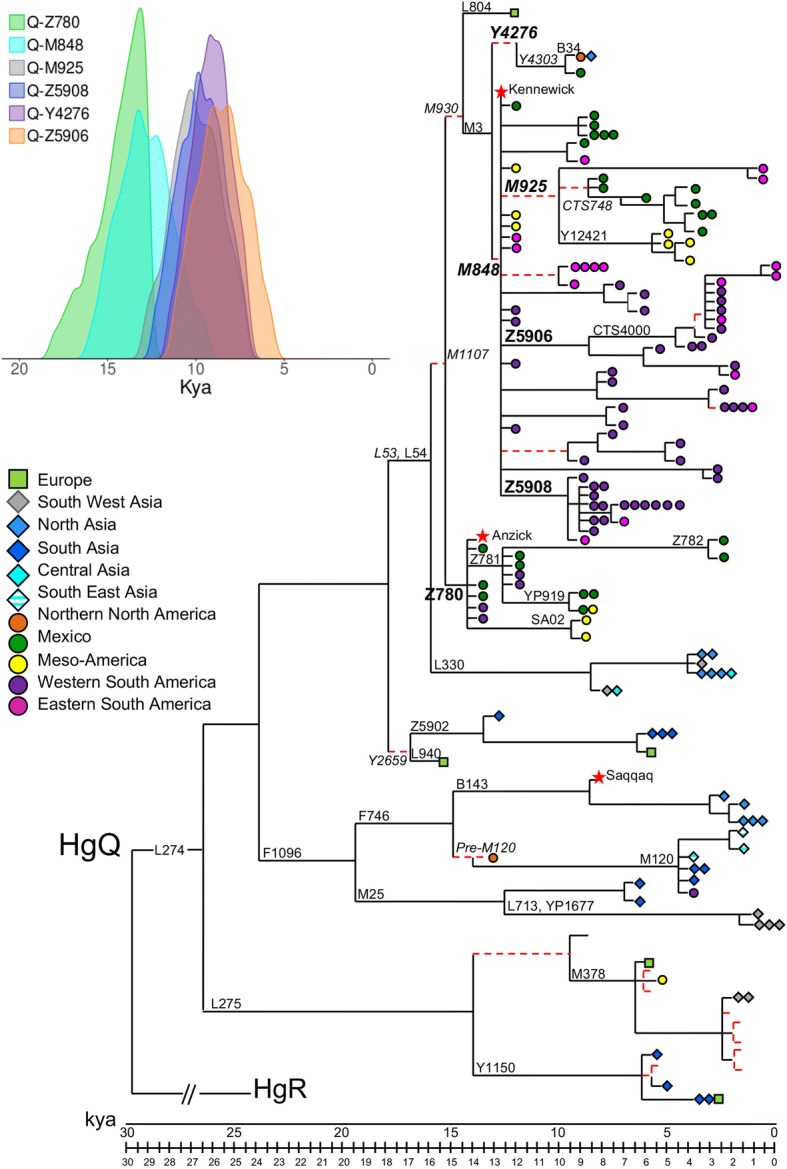
Condensed version of the most parsimonious (MP) tree of Y-chromosome haplogroup Q. This tree was obtained considering 1550 variable positions in 149 modern (this work; [22, 23, 26, 30, 39, 57–59, 61, 62]) and 3 ancient [6, 28, 58] Y chromosomes. Their geographic origin and Y-chromosome haplogroup affiliation are reported in Additional file 1: Table S1. The length of each branch is proportional to its age estimate. The name of the marker(s) defining the branches discussed in the text is shown. Markers reported in italics are outside the sequenced fragments, and the relative branches are reported as dashed red lines. Stars indicate ancient Y chromosomes, squares Europeans, rhombi Asians and circles Native Americas; colours of the symbols are according to the macro-areas defined in Additional file 2: Table S2. The 95% highest posterior density intervals for the Q-Z780, Q-M848, Q-M925, Q-Z5908, Q-Y4276 and Q-Z5906 TMRCAs are shown in the inset

The comparison of 154 Y-chromosome sequences (Additional file 1: Table S1), of which 34 new (Additional file 2: Figure S1), with the A00 sequence [22, 26], a member of the deepest branch of the Y-chromosome phylogeny, revealed 1550 nucleotide positions carrying a derived allelic state (1563 including recurrent positions, 1515 within haplogroup Q, Additional file 3: Table S2). Out of these, 826 (53.3%) variant positions were not annotated and 488 were also not described in ISOGG, YFull or in Karmin et al. [22]. Six variant positions were located within the coding regions of the *RPS4Y1*, *USP9Y*, *UTY* and *ZFY* genes (Additional file 4: Table S3) with a coding/non-coding variant ratio of 3.9 × 10^−3^, a value lower than the 8.1 × 10^−3^ previously reported [27]. The relationships between variants are illustrated in the phylogenetic tree of Fig. 1 and detailed in Additional file 5: Figure S2, where the ages of the identified sub-lineages are also provided. The tree incorporates informative SNPs outside the studied regions, which have become available from the literature [22, 23] and/or from genealogy websites (ISOGG tree; YFull tree). These SNPs are included in the list of variable positions (Additional file 3: Table S2) and shown in italics in the phylogeny of Fig. 1 and in Additional file 5: Figure S2.

The phylogeny reveals four main bifurcations identifying five main sub-haplogroups of Q: Q-L275, Q-F1096, Q-Y2659, Q-L330 and Q-M1107. Two sub-branches of Q-M1107, Q-Z780 and Q-M3, are American-specific and characterize the ancient remains of Anzick-1 and Kennewick, respectively; Q-F1096 harbours Asian and Arctic Native American samples as well as the Greenland ancient specimen belonging to the Saqqaq culture [28], while the remaining sub-haplogroups include only Eurasian Y chromosomes.

To investigate the distribution of the main clusters as well as the identified Native American sub-haplogroups, we performed a hierarchical genotyping of the main haplogroup-defining markers (Additional file 6: Table S4) in 409 modern samples of our dataset (320 Native Americans and 89 from Eurasia). The haplogroup classification, summarized in Additional file 7: Table S5, was combined with data available in the literature (Additional file 8: Table S6), and the geographic distribution of the most diffused haplogroups is illustrated in Additional file 9: Figure S3, Additional file 10: Figure S4, Additional file 11: Figure S5, Additional file 12: Figure S6, Additional file 13: Figure S7 and Additional file 14: Figure S8.

### The modern Eurasian branches of haplogroup Q and their link with proto-Native Americans

Q-L275 is the branch originated by the first bifurcation (before 26 kya; between 27.8 and 32.5 kya according to Poznik et al. [29]) of haplogroup Q. It comprises Q-Y1150 and Q-M378. The first is mainly observed in Southwest Asia with some appearances in Northwest Eurasia, while the second, recently dissected [30], is spread across West, Central and parts of South Asia and harbours mainly Middle Eastern Y chromosomes, with one branch typical of Ashkenazi Jews, as well as European samples (Additional file 9: Figure S3a,b). Based on this distribution, the two M378 Y chromosomes observed in the Isthmo-Colombian area (Additional file 9: Figure S3b) should be interpreted as the result of a post-Columbian arrival from Eurasia, as previously hypothesized [20].

Q-F1096 splits into Q-F746, which in turn includes Q-B143 and Q-M120, and into Q-M25. The distribution of Q-B143 both in Northeast Siberia, in the North American Arctic and in Greenland (the F746 Y chromosomes observed in the Athabaskans [19] and in different Greenland districts [31] are likely Q-B143) is in agreement with a Paleo-Eskimo dispersal of this lineage. Indeed, the Saqqaq Y chromosome, which belongs to this haplogroup, was dated ~ 4 ky [28], in the period of the first colonization of the North American Arctic accomplished by Paleo-Eskimos.

Q-M120, widespread in Southeast Asia, is observed in one South American subject while a pre-M120 chromosome was described in Alaska. According to the phylogeography (Additional file 9: Figure S3c) and STR haplotype variation of Q-M120 [32], the South American subject might be ascribed to a recent event of gene flow. This is not the case for the Alaskan Y chromosome, which stems from a precursor of M120 (hence named Q-pre-M120) dated 14.2 ± 2.1 ky.

Q-M25 is observed from Eastern Europe until Central Asia with its highest frequency in the Iranian Plateau where it is virtually only represented by its clade Q-L712 (Additional file 9: Figure S3c). This lineage has not been observed in present-day North Native Americans, but it has been recently reported in ancient Aleutian Islanders, ancient northern Athabaskans and in a 4250-year-old individual of the Chukotkan Ust’-Belaya culture [33].

Q-Y2659 (Additional file 10: Figure S4a) was not observed in America. It includes two branches, the first, Q-Z5902, mainly observed in South Asia while the second, Q-L940, in Western Eurasia. The latter includes a Northwestern European sub-lineage, Q-FGC7000, observed also in a Sardinian subject. Although this could be interpreted as the result of a recent gene flow event, it could also represent a relic of the ancient migrations that brought the first settlers on the island as for Y-chromosome haplogroup G-L91 [34] and some mtDNA haplogroups [35, 36].

### Q-L53: the phylogenetic crossroad of Asian-, European- and American-specific branches

Q-L53(xL54) Y chromosomes have been described in Central Asia [20, 37]. Recently, Q-L53(xL54) Y chromosomes have been reported in five Eastern European subjects as belonging to the new clade Q-YP4004 (Additional file 10: Figure S4b, YTree v6.02 - https://www.yfull.com/tree/Q/). These findings suggest that also the Central Asian Q-L53(xL54) Y chromosomes described above [37] belong to the Q-YP4004 branch. Interestingly, one ancient specimen from the Lovelock Cave in Nevada dated 1.8 ky [38] falls in this clade representing the only evidence of this lineage in America. Therefore, L53 likely originated in Central Asia and, before spreading, differentiated into Q-YP4004 and Q-L54.

Q-L54 includes Q-M1107, which encompasses the European Q-L804 and the Native American clades Q-Z780 and Q-M3 (Fig. 1; Additional file 10: Figure S4b,c) and the Eurasian branch Q-L330 [19].

The Q-L330 branch is mainly diffused in Central East Asia with few representatives in Western Eurasia (Additional file 10: Figure S4b). The age of its MRCA has been estimated at 8.3 ± 1.5 ky, much younger than the bifurcation that separates this lineage from Q-M1107 (15.6 ± 1.8 kya). However, the estimate is based essentially on East Asian subjects belonging to only one of the two L330 branches; therefore, the value might be biased and the actual Q-L330 MRCA could predate the estimated age. The lower frequencies of Q-L330 compared to its sister clade Q-M1107 suggest that Q-L330 underwent a strong bottleneck somewhere in Central Asia before spreading in Eurasia.

Q-L804 is represented in our phylogeny by one English Y chromosome previously identified as sharing a deep node with Q-M3 [39]. This finding is in full agreement with the Q Nordic project of FTDNA (http://hoijen.se/2016/01/29/q-l804-current-status-2016-01-29/) that has detected this rare and apparently European-specific lineage in English, Norwegian and French participants (Additional file 10: Figure S4c). These results support an Asian origin of M930, the upstream marker of L804 and M3, a North Eurasian route of dispersal and a recent dissemination in some North European populations.

### The Native American-specific branches Q-Z780 and Q-M3 and their sub-clades

Q-M3 and Q-Z780 are the two main Y-chromosome founding lineages of Native Americans. Both have been observed in ancient American DNAs: the Anzick-1 Y chromosome, which is dated at 12.6 ky [40] and belongs to Q-FGC47532, a sub-branch of Q-Z780, and the Kennewick Y chromosome, dated at 9.0 ky [41], which falls into Q-M848 (Fig. 1). Although Q-Z780 coalesces at 14.3 ± 1.6 ky and Q-M848 at 12.5 ± 1.6 ky, their simultaneous arrival in Central America [20] suggests their presence in the first Native American founding population groups. On the other hand, the lower age of Q-M848 (see also its 95% highest posterior density interval—Fig. 1, inset) could be biased by the age of the great expansion that this branch underwent.

Inside *Q-M3*, the two branches *Q-Y4276* and *Q-M848* are distinguishable.

Despite its poor representation in the tree (Fig. 1), *Q-Y4276* displays the widest geographic distribution, being observed from Siberia to South America (Additional file 11: Figure S5). It is the main clade found in the USA (in Virginia, Carolina and Georgia but also in the South West) where it is present as Q-Y4276*, Q-Y4300 and Q-Y4303* and seems to be associated with subjects speaking Algonquian language (http://haplogroup.org/native-american-q-m3-tree-p2-q-m242-news-6-nov-2016/). Algonquian is one of the most populous and widespread North American Native language groups. Historically, it was prominent along the Atlantic Coast and into the interior along the St. Lawrence River and around the Great Lakes and the Rocky Mountains. In North America, the distribution of the Y-chromosome lineage Q-Y4276 parallels that of mtDNA haplogroups X2a and the rare C4c (Additional file 15: Figure S9) [11], which have been postulated to have entered from Beringia into North America through the ice-free corridor between the Laurentide and Cordilleran ice sheets [9, 10, 14]. Thus, it is possible that the same groups carrying the mtDNA haplogroups X2a and C4c might have brought Q-Y4276 Y chromosomes in North America. Moreover, the “Northern Native American” or “Ancestral B” (ANC-B) component identified in several ancient Native American genomic studies [4, 5, 38, 42] displays a similar pattern.

Interestingly, the observation of Q-Y4303* in a single Brazilian sample is reminiscent of an analogous finding, i.e. the rare mtDNA haplogroup C4c in Colombia [14], and supports the scenario that genomic ANC-B might have contributed to the Central and South American gene pool [5], either through an ancient migration of this lineage or through a recent contact with the Northern sub-continent. The well-differentiated position of the Brazilian sample in the network of the STR haplotypes associated with this haplogroup (Additional file 11: Figure S5) seems to support the first scenario. If this interpretation is correct, the finding of this lineage in Brazil, but not in the numerous samples from the western regions, would suggest that it entered South America through the Atlantic coastal route [15] instead of the Pacific coastal route [16]. The MRCA of this clade has been dated 9.3 ± 1.2 ky, thus extensively post-dating the peopling of the Americas; however, its STR variation evaluated on a wider dataset would place its age estimate in the time frame of the first entry into the double continent.

*Q-M848* is the most represented branch of haplogroup Q in the Americas both in modern and ancient times (Additional file 12: Figure S6, Additional file 13: Figure S7 and Additional file 16: Figure S10). *Q-M925* is the most diffused of its sub-haplogroups, with samples from the USA to South America. It includes four branches (Q-Z4012, Q-Y26547, Q-Y12421 and Q-CTS748) with specific geographic distributions and some still not sub-classified Y chromosomes (M925*). *Q-Y26547* is found in two Brazilian samples; *Q-Y12421* is observed both in Mexico and the Southwest USA and characterizes the majority of Panamanian M3 Y chromosomes; *Q-CTS748* encompasses almost all the Mexican M3 Y chromosomes of our dataset, with half of these further characterized by the CTS1002 marker. In addition, it is also sporadically observed in the Southwest USA (https://haplogroup.org/native-american-q-m3-tree-p2-q-m242-news-6-nov-2016//). The age estimate of Q-M925 is 9.8 ± 1.4 ky (Additional file 12: Figure S6; inset of Fig. 1). Among the three branches tested in this study, the Mexican Q-CTS748 turned out to be the most ancient (8.5 ± 1.4 ky), followed by its sub-clade Q-CTS1002 (6.8 ± 1.2 ky). The remaining two branches are much more recent (Q-Y12421: 5.3 ± 1.0 ky; Q-Y26547: 1.2 ± 0.6 ky) although their age estimates could be biased by the very small number of samples included in the tree. Network analyses of the STR haplotypes (Additional file 12: Figure S6), associated not only with Q-CTS748 but also with Q-Y12421, reveal an internal high complexity of these clades, suggesting the presence of unidentified sub-clades as well as older ages.

*Q-Z5906* and *Q-Z5908* display similar distribution patterns (Additional file 13: Figure S7) from Mexico to Argentina with a frequency peak in Peru where both show the greatest diversification, each one including a local specific sub-clade: Q-M557 and Q-SA01, respectively. Q-Z5906 is almost completely represented by CTS4000, present at high frequency also in Bolivia (Additional file 13: Figure S7a). As shown in the network of the STR haplotypes, a clear expansion of all Z5906 sub-lineages is detected in Peru and Bolivia. Conversely, Q-Z5908 (Additional file 13: Figure S7b) displays an earlier differentiation without any sign of expansion of its sub-lineages despite being well represented in that area. The differences observed for these two clades could be due to dissimilar subsistence behaviours and demographic dynamics over time of their carriers.

As for *Q-Z780* (Additional file 14: Figure S8), the present level of resolution distinguishes three main groups of Y chromosomes: Q-Z781, Q-SA02 and Q-Z780*(xZ781, SA02). The first is the most represented and structured as well the oldest (12.5 ± 1.5 ky). Its dating indicates that it is nearly coeval with lineage Q-FGC47532 characterizing the Anzick-1 Y chromosome (^14^C dated at 12.6 kya). However, the microsatellite variation associated with Q-Z781 suggests an older age. Q-Z781 includes three sub-lineages, Q-YP910, Q-Z782 and Q-YP919. While Q-YP910, represented in the tree by only one subject, cannot be dated, Q-Z782 and Q-YP919 turned out to be 3.1 ± 1.1 ky and 9.6 ± 1.4 ky old, respectively. Q-YP919 includes two Mesoamerican expansions, marked by the BZ1716 and M4743 mutations, well defined in terms of STR variation and dated to about 5 kya (Additional file 14: Figure S8). Another Mesoamerican contemporaneous expansion event is also registered by Q-YP910. Q-SA02, which is represented by few samples and dates back to 9.3 ± 1.5 kya, seems to be restricted to the Isthmo-Colombian Area. Chromosomes Z780*(xZ781, SA02) are observed both in Mexico and in the Andean region and are characterized by a wide variation and a complexity still to be resolved as shown in the network of their STR haplotypes (Additional file 14: Figure S8), where at least two highly variable sub-lineages are visible, suggesting an ancient origin of their MRCA.

### Bayesian analyses: different regional population growths after the initial expansion of Q-M1107 into the Americas

**Fig. 2:**
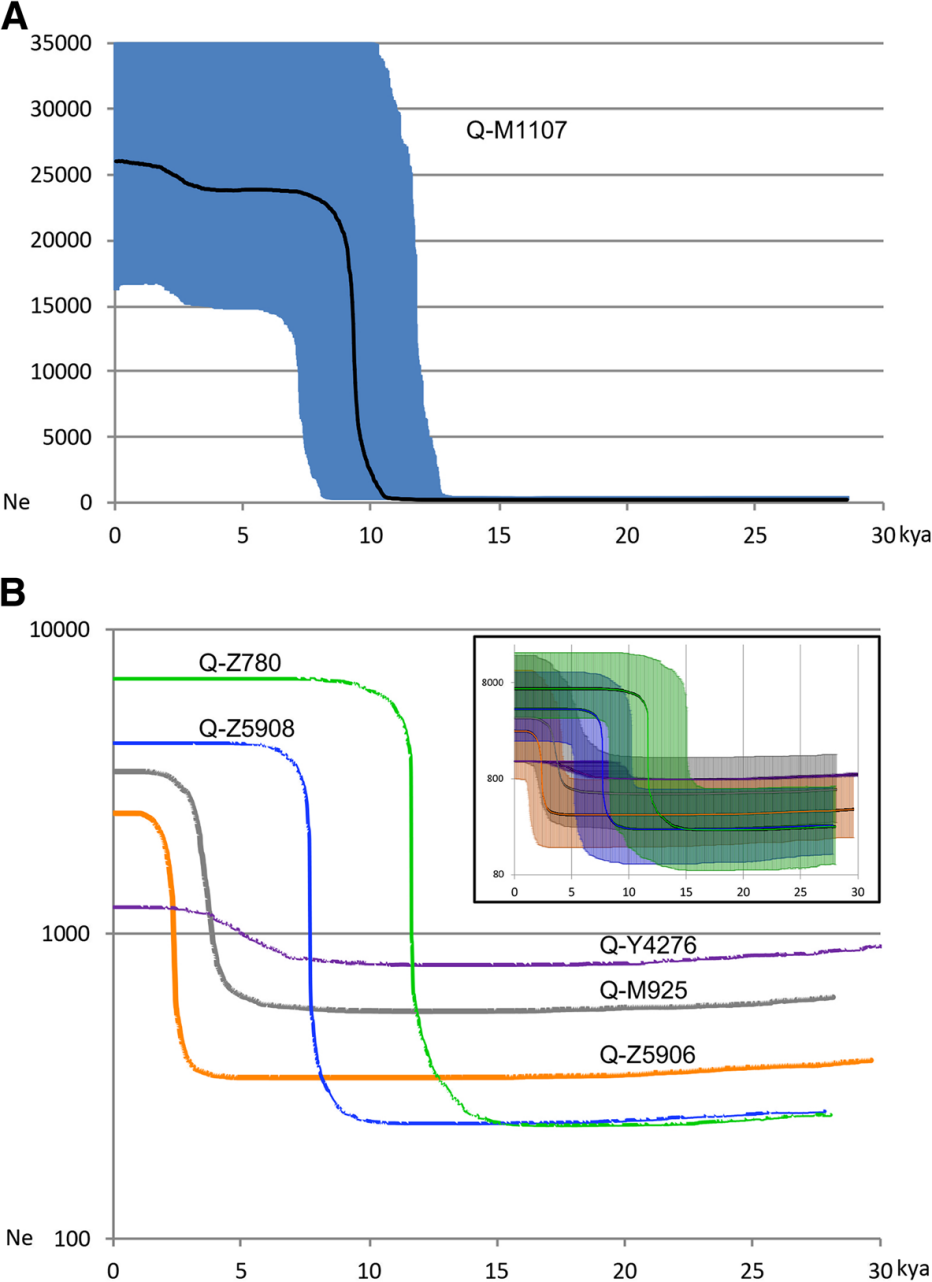
Bayesian skyline plots (BSPs) of Q-M1107 lineage and its main sub-lineages in Native America. Bayesian skyline plot (BSP) depicting the population size changes through time in the Americas for the 1.5 Mb of the Q-M1107 Y chromosomes included in the phylogenetic tree of Fig. 1. All Native American M1107 Y chromosomes were considered together in panel **a**, while only those belonging to the main sub-lineages were assessed separately in panel **b**. Timing of events was estimated on the basis of Kennewick’s and Anzick-1’s radiocarbon dates [40, 41]. The *x*-axis is in calendar thousand years before present, and the *y*-axis equals changes in effective population size (shown as the product of *N*_*e*_ and generation time *T*). The black and coloured lines in panel **a** and in panel **b**, respectively, are the median estimates while the shadings in panel **a** and in the inset of panel **b** show 95% highest posterior density intervals. The time axis is limited to 30 kya, beyond which the curve remains flat

Through a Bayesian method, the posterior distribution of the effective population size through time was estimated for the entire sample of Native Americans and, separately, for the samples belonging to the most significant sub-haplogroups described above (Fig. 2). The analysis of the entire Q-M1107 (Fig. 2a) shows a major phase of population growth after 15 kya followed by a period of constant population size from 8 until 3 kya when another slight sign of general population growth is apparent. Taking into account that only few samples were from North American Natives, the first part of the curve could be mainly ascribable to two important growth events. The first revealed by haplogroup Q-Z780 (green curve in Fig. 2b) started around 15 kya in Mesoamerica and in the Isthmo-Colombian Area, in agreement with an entrance in South America prior to 15 kya [43]. The second one, registered by haplogroup Q-Z5908, occurred during the Holocene in the western part of South America (Peru) and was probably associated with the improvement of the climatic condition [44]. This is the period when the domestication of cassava, pumpkin and sweet potato slowly started in the region [45]. Afterwards, American population size remained constant until 3 kya when the second period of growth started. This scenario is also supported by the hints of growth of Q-M925 (grey in Fig. 2b) mainly in Mesoamerica (Additional file 12: Figure S6) and of Q-Z5906 (orange curve in Fig. 2b) mainly in Peru (Additional file 13: Figure S7). A similar trend emerges also from archaeological data in South America: a first signal of growth linked to a resource-limited (megafauna extinction) growth over time was followed in North West South America by about 9 ky of slow domestication until 3 kya when cultural and technological changes occurred [46] causing a shift to a predominantly sedentary and agricultural subsistence with the consumption of maize and sweet potato [45, 47]. What appears from these reports is that changes in South America were isolated and different for each population, due to divergent environments and geographic barriers, hence not able to support a single cohesive culture diffusion as in Europe [48].

## Discussion

### The peopling of America: considerations from the dissection of Y-chromosome haplogroup Q

The Asian origin of Native Americans is well established, and several migration models and entry scenarios have been proposed (mostly by analysing uniparental markers) to account for the variation observed in modern Native Americans [9, 10, 12]. In the last years, genome studies on modern and ancient samples have confirmed the Asian origin of Native Americans [2] indicating that (i) present-day Natives descend from at least three distinct ancient waves of migrations, the first along the double continent and the other two in northern North America involving Paleo- and Neo-Eskimo populations around 5 kya and 1 kya, respectively; (ii) the main contribution derived from groups of people that separated from the ancestors of present-day East Asians more than 20 kya and that settled in Beringia for several thousand years, before moving into North and South America; and (iii) two main components, one in North Native Americans and one shared by Central and South American peoples, were distinguishable [28, 40, 41, 49, 50]. The northern branch would be ancestral to populations including the Algonquian, Na-Dene, Salishan and Tsimshian speakers of Canada and likely the ancient Kennewick while the Southern branch would include the ancestors of all Native Americans from Mexico and Central and South America as well as Anzick-1. The recent sequencing of one 11.5-ky-old genome from Siberia (USR1) allowed to identify a distinct ancient Beringian population closely related to Native Americans but basal to all previously sequenced contemporary and ancient Native Americans [4]. Subsequently, the information derived from the genome sequencing of a large number of ancient samples from two areas of North America occupied by modern humans since 13 kya, the Channel Islands of California and Southwestern Ontario, confirmed the presence of two basal ancestries (ANC-A and ANC-B) [5, 38]. These two components turned out to be equally distant from the Siberian USR1 sample but, in contrast to previous proposals, to be unevenly represented in Central and South America. To explain these observations, the authors proposed a split of the ancient Siberian population followed by some thousand years of differentiation of the two branches in North America and subsequent multiple events of admixture [5]. However, the time and the place of this split (within North America [5], Northeast Asia or Eastern Beringia [4]) and the routes followed by the carriers of the two components are still under evaluation/discussion [4, 5, 17, 38, 42].

**Fig. 3:**
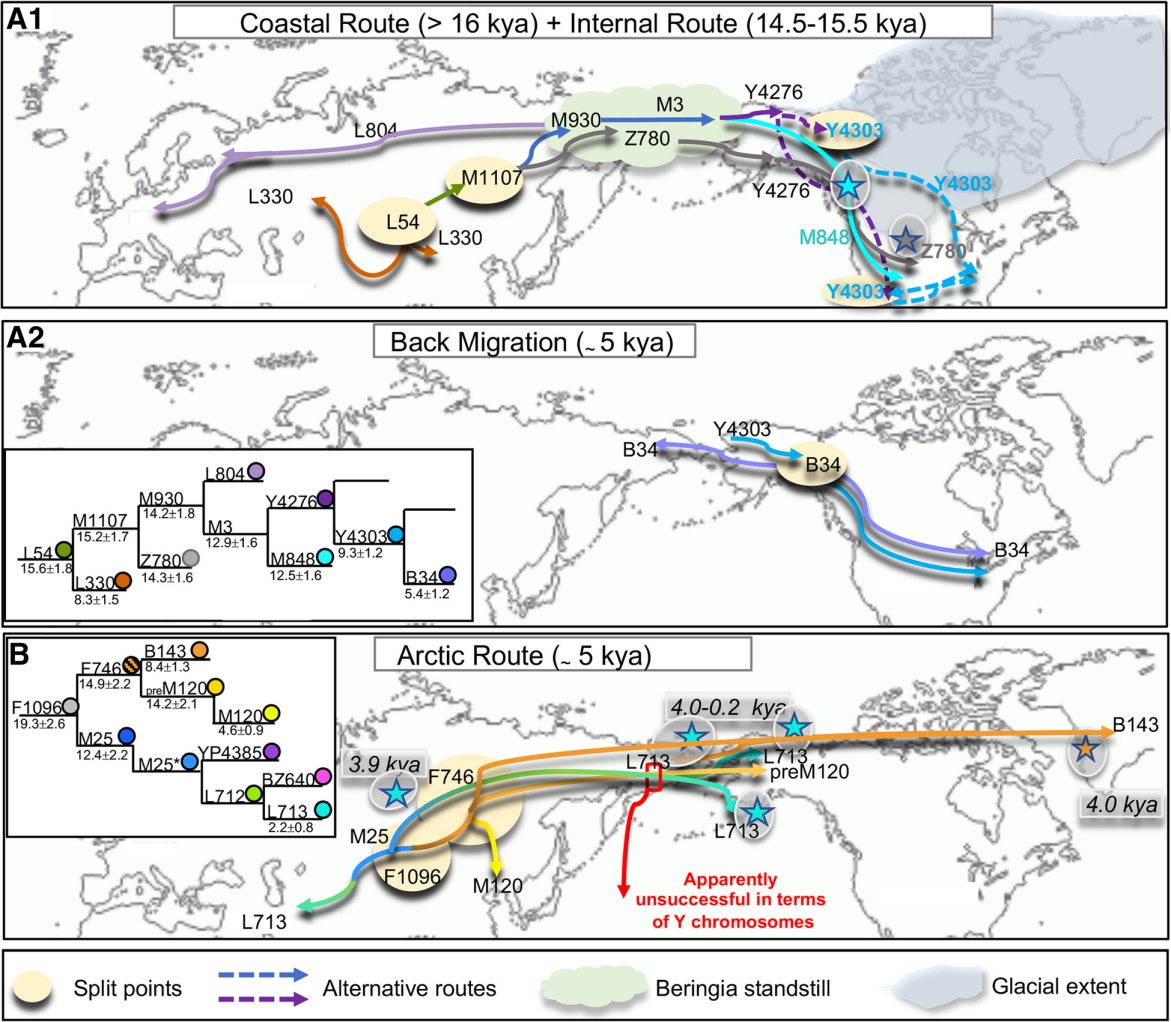
Main migratory events from Beringia/Asia towards North America and back migrations according to the Y-chromosome variation. Schematic representation of the spread of the Q-L54 sub-lineages Q-L804 and Q-L330 in Eurasia and of Q-Z780, Q-M848 and Q-Y4276 from Beringia to North America (**A.1**) and back migration to Asia of Q-B34 (**A.2**). For Q-Y4276, we propose two possible entry routes (dashed lines) into North America. **B** Schematic representation of the spread of the Q-F1096 sub-lineages Q-L713 and Q-M120 in Eurasia and of Q-L713, Q-preM120 and Q-B143 in Arctic North America. While Q-pre-M120 and Q-L713 mark migrations towards North America apparently unsuccessful in terms of Y chromosomes, Q-B143 reached Greenland. The presence of recent Q-B143 Y-chromosomes in the Koryaks of Siberia can be explained by a back migration. The two insets illustrate the phylogenetic relationships of the Q sub-lineages (in different colours). Stars highlighted by a grey shading refer to ancient samples

Our study, focused on Q, the only Pan-American Y-chromosome haplogroup, confirms the Asian origin of Native Americans and provides information about the main Asian-American migrations as well as some unsuccessful migration attempts (in terms of Y-chromosomes) and some back migrations (Fig. 3). *Q-M1107*, which encompasses the majority of Native American Y chromosomes, provides information about the first peopling of the Americas. One of its branches, *Q-Z780* (previously known as Q-L54*(xM3, M330)) is observed only in America whereas the other, *Q-M930*, encompasses both the Pan-American *Q-M3*, which is also found only in the Americas and the Northwest European *Q-L804*. Thus, it is likely that Q-Z780 and Q-M930 were both present in the ancestral Asian/Beringian source population that gave rise to Native Americans.

Q-Z780, whose age estimate seems to overlap (14.3 ± 1.6 ky) the melting of ice sheets (14.5–15.5 kya), most likely was carried by the first settlers of the double continent and moved rapidly southward following the Pacific coastal route. It characterizes Anzick-1 Y chromosome (12.6 ky) in North America and four ancient specimens (8.3–3.3 ky) in South America [42].

Q-M930 differentiated into Q-L804 and Q-M3 during the Beringian standstill [51]. From Beringia, carriers of the first branch moved westwards reaching North Europe while Q-M3 entered the American continent where it further differentiated into *Q-M848* and *Q-Y4276*. Q-M848, whose variation is only slightly lower (12.5 ± 1.6 ky) than that of Q-Z780 (Fig. 1), likely moved also southward along the Pacific coast probably together with the “Southern Native American” or “Ancestral A” (ANC-A) component [5, 38, 42].

In Central America, both Q-Z780 and Q-M848 show clades older than 10 ky, confirming the rapid and nearly simultaneous arrival in the region. In South America, only Q-M848 is well represented, displaying different potentially “area-specific” clades with coalescent times around 8.0 ky. Q-M848 characterizes almost the totality of the ancient samples (Additional file 16: Figure S10) from North America [4, 28], the Californian Islands [5] and from Patagonia [52].

As for M3, the second Native American-specific branch, it is likely that its Q-Y4276 sub-branch arose in Beringia, evolving early into Q-Y4303 in northern North America as suggested by its estimated age (9.3 ± 1.2 ky) (Fig. 3A.1). From there, the population(s) carrying these markers migrated southward spreading Q-Y4303 in the southern part of California and in Mexico and the sub-branch Q-Y4300 to the eastern part of northern North America where it characterizes Algonquian groups (Additional file 11: Figure S5). The few Q-Y4300 subjects observed in Southwest USA/Mexico might represent the legacy of Southern Athabaskans. An early differentiation in northern North America of Q-Y4303 would also explain the distribution of its sub-branch Q-B34 in the northern area of North America as well as its presence in two ancient samples from Alaska and Quebec [5]. On the other hand, the presence of Q-B34 (5.4 ± 1.2 ky) also in the Koryak of Siberia can be easily attributed to a back migration (Fig. 3A.2), as postulated for mtDNA haplogroup A2a [11].

Q-F1096, which is diffused in Asia where it comprises *Q-M25* (dated 12.4 ± 2.2 ky) and *Q-F746* (dated 14.9 ± 2.2 kya), provides information about ancient movements towards Beringia and the Eskimo diffusions into the Arctic regions of North America (Fig. 3B). Indeed Q-M25, which is frequent in modern Western Eurasians [53, 54], is described as Q-L712 and Q-L713 in ancient samples from the Beringian area [33] indicating that, during the warmer mid-Holocene period, populations carrying different haplogroup Q lineages reached the former Beringian area but gave a limited contribution to the modern Y chromosome gene pool. Differently, *Q-F746*, which is common in Southeast Asia as Q-M120 (4.6 ± 0.9 ky), encompasses the pre-M120 lineage (dated 14.7 ± 2.3 ky) observed in an Alaskan subject (Tsimshian), the Q-B143 lineage (8.4 ± 1.3 ky), which characterizes the Saqqaq Paleo-Eskimo (4 ky), and the new branch Q-PV706 (2.8 ± 0.9 ky) observed in a few Koryaks of Northeastern Siberia. Thus, the Alaskan pre-M120 might represent a relic of the East Asian contribution to the ancient Siberian population involved in the first peopling of the Americas that, differently from Q-Z780 and Q-M848 lineages, did not have success.

Q-B143 would trace the Paleo-Eskimo migration at around 4 kya; in this scenario, the Q-F746 Y chromosomes observed in the North America Arctic [19] and not yet assessed for B143 could include both Paleo- and Neo-Eskimo contributions to the Arctic people; in turn, the lineage Q-PV706 observed in the Koryaks might represent either an East Asian evolution of Q-F746 or a back migration from North America as for Q-B34.

During the long standstill in Beringia of the ancestors of Native Americans, the Beringian gene pool was characterized not only by the two M1107 branches, Q-M930 and Q-Z780, but probably also by Q-F746 as a precursor of Q-pre-M120 and Q-B143. Prior to their entry into the Americas with the first settlers, both Q-Z780 and Q-M3 underwent further differentiation and genetic drift. Q-F746 instead does not appear to have participated to the first peopling of America: the pre-M120, which still persists in the Tsimshian population of Alaska, was apparently unsuccessful; Q-B143 must have survived in Siberia in ancestral Eskimo populations until its diffusion in the North American Arctic after 5 kya. Thus, with regard to the first peopling, the split of Q-M3 into Q-M848 and Q-Y4276 could correspond to the separation of the two main population groups. In this scenario, Q-M848 and Q-Z780 would have been carried along the Pacific coast by the population group that gave rise to the ANC-A component, whereas Q-Y4276 could have followed the internal route as Q-B34 and Q-Y4300 contributing, together with the mtDNA haplogroups X2a and C4c (Additional file 15: Figure S9), to the component (ANC-B) that mostly appears to characterize northern Native Americans. In such a scenario, taking also into account the back migration of Q-B34, the split of the populations ancestral to ANC-A and ANC-B would be best placed in eastern Beringia prior to their entry into America. On the other hand, new data [3] indicate that ice-free corridor was viable much earlier than previously thought (15.6–14.8 kya), thus reviving the possibility of distinctive migration paths of the ancestral Native American components. The observation that the Kennewick genome, which carries mtDNA haplogroup X2a and belongs to the ANC-B component, is characterized by the Y-chromosome haplogroup Q-M848 suggests also that the following (recently proposed) admixture events [5] must have started very early in North America.

## Conclusions

In conclusion, we established and dated a detailed haplogroup Q phylogeny that provides new insights into the geographic distribution of its Eurasian and American branches in modern and ancient samples.

For the first time, we found two distinct Y chromosome lineages mirroring the two main “ancestral” components (ANC-A, ANC-B) previously characterized by recent genomic studies [5, 38, 42]. The differentiation of these lineages likely occurred in eastern Beringia before their entry into America following two routes: the coastal route (ANC-A, Q-Z780/Q-M848) and the internal route (ANC-B, Q-Y4276). Once entered America, these two ancestral components probably admixed very early in North America as suggested by the ancient Kennewick nuclear genome belonging to ANC-A (Q-M848) yet carrying an ANC-B mtDNA haplogroup (X2a).

Moreover, we traced two major expansions of the ANC-A lineages in Meso- and South America, one around 15 kya, early after the first peopling, and another at 3 kya, following climatic improvements and local cultural transitions.

Further support to our conclusions and new insights might come from the analysis of Native modern and ancient genomes spanning the entire temporal frame of first America’s peopling, including currently underrepresented regions of the continent.

## Methods

### The sample

Thirty-four unrelated males, 25 from Meso- and South America [20] and 9 from Asia, the Middle East and Europe [53, 55, 56], were high-depth re-sequenced for a large portion (3.7 Mb) of the MSY [27]. To increase the chance of identifying new phylogenetically informative SNPs, the samples were selected taking into account their geographic origin and Y-STR haplotypes. One hundred eighteen Hg Q MSY Native American sequences were from the literature: 115 from modern samples [6, 22, 23, 30, 39, 57–60] and 3 from ancient DNA specimens (“Saqqaq”, “Anzick-1” and “Kennewick”) [28, 40, 41, 61].

### Phylogeny

Phylogenetic analyses were carried out on 152 male samples belonging to haplogroup Q (Additional file 1: Table S1) from 14 distinct geographic regions (Additional file 2: Figure S1). Two sequences belonging to Hg R1b, the sister clade of haplogroup Q, were also included in the analyses [26, 62].

### Phylogeography

Geographic distributions of the sub-haplogroups were evaluated by analysing 1549 modern Y chromosomes belonging to haplogroup Q: 711 Native Americans and 838 from Eurasia. Among these, 320 Native American and 89 Eurasian Y chromosomes were sub-classified in this study, while the remaining were from the literature or specialized websites (Additional file 8: Table S6).

### Deep re-sequencing and sequence analysis

On the whole, five regions of the X-degenerate portion of the MSY were sequenced for a total of 3,768,982 bp for each Y chromosome. Of these, 1,495,512 bp were considered for the analysis, representing 5274 unique fragments [27]. The employed capture design includes six protein-coding genes (*RPS4Y1*, *ZFY*, *USP9Y*, *DDX3Y*, *UTY* and *TMSB4Y*). Library preparation, targeting, sequencing and alignment were performed by BGI-Tech (Shenzhen, China) as previously described [27]. BAM files were visually inspected using Integrative Genomics Viewer (IGV) [63, 64]. Variants were filtered out using SAMtools [65] and BCFtools. GASVPro [66] and BreakDancer-v 1.1 [67] were used to verify, a posteriori, that structural rearrangements did not cause clustering of variants in short stretches of DNA. For all positions found as variants in at least 1 of the samples considered, and included in the above mentioned 5274 fragments, the read depth and the quality score of consensus (QS) were investigated in the 34 re-sequenced samples. Two main criteria were used to assess the validity of a candidate mutation: QS > 90 and a difference between the depth and the total number of reads for the two best bases ≤ 4. Variant calls with QS = 99, depth ≥ 4 and a difference < 1 were considered true mutations. Variant calls with QS ≤ 90 or QS > 90 but a difference > 4 were indicated as not available (NA) to discard potential false SNP calls, while calls with 90 ≤ QS ≤ 99 and 1 ≤ difference ≤ 4 were manually inspected. Detailed information is provided in Additional file 17

### Parsimony tree construction and time estimates

A median-joining network and a maximum parsimony (MP) tree were constructed on the basis of a list of variable positions per subject, by using Network 5.0 [68] and MEGA6 [69], respectively. The median-joining network provided a complete listing of mutated positions along each branch and a precise count of inferred recurrent mutations (.out file), which were re-checked and confirmed in the original alignment files. The haplogroup Q phylogenetic tree and the coalescent times of its sub-haplogroups were estimated through the software BEAST 1.8.3 [70]. We employed a coalescent expansion growth model for the population size, the radiocarbon dates of “Kennewick” (9.0 ± 0.1 ky), “Anzick-1” (12.6 ± 0.1 ky) and “Saqqaq” (4.0 ± 1.0 ky) as tip dates, a strict clock rate and rather flat priors for the current population size (lognormal[3,10]) and for the ancestral/current population size ratio (exponential[0.2]). Two runs of 20 million steps each, sampled every 10,000 steps, were performed.

Trees reconstructed under this model were combined after discarding the first 200 generations of each replicate as burn-in using LogCombiner v.1.8.3 (http://beast.community/logcombiner). The trees were summarized using TreeAnnotator v.1.8.3 (http://beast.community/treeannotator), and the results of divergence times were visualized on a maximum clade credibility (MCC) tree produced using FigTree v.1.4.2 (http://tree.bio.ed.ac.uk/software/figtree). The highest posterior densities (HPD), that collect the most probable age distributions, were calculated for each TMRCA considered, taking into consideration the effective sample size (ESS) parameter. Coalescent time estimates were obtained by combining .log files after discarding the first 2000 generations of each replicate as burn-in, and the results were visualized in Tracer v1.6 [71].

Bayesian skyline plots (BSP) were generated with Tracer v1.6 [71] using input from the files generated in the previous BEAST analyses. We employed for the analysis a coalescent Bayesian skyline tree prior, a GTR substitution model, and the radiocarbon dates of “Kennewick” (9.0 ± 0.1 ky) and “Anzick-1” (12.6 ± 0.1 ky) as tip dates. Two independent analyses of 20 million generations sampled every 10,000 were computed. Convergence was confirmed by effective sample sizes over 200 for both runs.

### Age from microsatellite variation

Ages based on microsatellite variation within binary haplogroups were defined by the methodology of Zhivotovsky et al. [72] as described in Karachanak et al. [73]. Microsatellite haplotypes are reported in Additional file 8: Table S6. It is worth mentioning that ambiguities related to past episodes of population history (e.g. size fluctuations, bottlenecks) create inherent uncertainties in the calibration of the Y-STR molecular clock; thus, the estimated ages of microsatellite variation should be considered with caution.

### Genotyping

Signature markers of 41 sub-haplogroups were defined (Additional file 6: Table S4). The sequences surrounding each variant position were downloaded from the UCSC DAS server. PCR primers were either designed with Primer3 software and checked with Primer-BLAST or chosen from YSEQ DNA shop (www.yseq.net). The genotyping was performed by Sanger sequencing or RFLP analysis.

## Additional files

Additional file 1: **Table S1.** List of the high coverage samples analysed in this study along with their geographic origin and Y-chromosome haplogroup affiliation. (XLSX 20 kb)

Additional file 2: **Figure S1.** Geographic origin of the subjects included in the phylogenetic analysis and listed in Additional file 1: **Table S1.** Circles and squares indicate modern samples from this study and from the literature, respectively. Stars indicate ancient samples: a-Kennewick; b-Anzick-1; c-Saqqaq. Colours identify different geographic macro-areas. When more than one subject is from the same area, the number of subjects is reported inside the symbol. (PDF 2653 kb)

Additional file 3: **Table S2.** List of variable positions defining the branches of the tree shown in Fig. 1 and Additional file 5: **Figure S2.** Branch nomenclature is according to Additional file 5: **Figure S2.** (XLSX 167 kb)

Additional file 4: **Table S3.** List of mutations in protein-coding regions of Y-chromosome genes. (XLSX 9 kb)

Additional file 5: **Figure S2.** Detailed version of the most parsimonious (MP) tree represented in Fig. 1 and estimated ages of the identified sub-haplogroups. Notes: The length of each branch is not proportional to its age estimate. For each branch, the name of the defining marker(s) and the number of mutations are reported. Markers in italics are outside the sequenced fragments and the relative branches are dotted. Nomenclature in blue colour is according to Jota et al. [25] while names in parentheses are according to Poznik et al. [29]. (a) Sample information in Additional file 1: **Table S1.** (PDF 962 kb)

Additional file 6: **Table S4.** Haplogroup signature markers analysed in the present study. (XLSX 14 kb)

Additional file 7: **Table S5.** Sub-haplogroup classification of Y chromosomes belonging to Q samples analysed in this study. (XLSX 15 kb)

Additional file 8: **Table S6.** Samples considered for phylogeographic surveys. (XLSX 212 kb)

Additional file 9: **Figure S3.** Phylogeography of the branches Q-Y1150, Q-M378 and Q-F1096 (panels a, b and c, respectively). Each panel illustrates the phylogenetic relationships of the markers (in different colours) investigated per each branch and their pattern of frequency distribution (complete list of samples in Additional file 8: Table S6). Circles without any number refer to one subject. Larger circles refer to the number of specified subjects. Stars highlighted by a grey shading refer to ancient samples: (1) [74], (2) [75], (3) [76], (4) [28]; their relative dating, when available, is also reported in italics. Dates reported below branches refer to Bayesian estimates of node ages. (PDF 1420 kb)

Additional file 10: **Figure S4.** Phylogeography of the branches Q-Y2659, Q-L53 and Q-L804 (panels a, b and c, respectively). Each panel illustrates the phylogenetic relationships of the markers (in different colours) investigated per each branch and their pattern of frequency distribution (complete list of samples in Additional file 8: Table S6). Circles without any number refer to one subject. Larger circles refer to the number of specified subjects. Stars highlighted by a grey shading refer to ancient samples: (1) [75]; (2) [38]; (3) [42]; their relative dating is also reported in italics. Dates reported below branches refer to Bayesian estimates of node ages. (PDF 1183 kb)

Additional file 11: **Figure S5.** Phylogeography of the Native American branch Q-Y4276. The panel illustrates the phylogenetic relationships of the markers (in different colours) and their pattern of frequency distribution (complete list of samples in Additional file 8: Table S6). Circles without any number refer to one subject. Larger circles refer to the number of specified subjects. Stars highlighted by a grey shading refer to ancient samples: 1) 523a, Alaskan Athabaskan; 2) RM-85, 618–518 ya; 3) SN-11 and SN-38, Late San Nicolas, 1172 ± 39 ya [5]. Dates reported below branches refer to Bayesian estimates of node ages. The network of the available STR haplotypes (Additional file 8: Table S6) and the estimated age associated with this clade are also shown. (a) Q-M3 samples not better sub-classified that could belong, at least in part, to Q-Y4276 [19]. (PDF 312 kb)

Additional file 12: **Figure S6.** Phylogeography of the Native American branch Q-M925 and its sub-branches. The panel illustrates the phylogenetic relationships of the markers (in different colours) and their pattern of frequency distribution (complete list of samples in Additional file 8: Table S6). Circles without any number refer to one subject. Larger circles refer to the number of specified subjects. Q-BZ4012 has been reported in YFull tree (YTree v6.02 - https://www.yfull.com/tree/Q/) where it is represented by a North Native American Y chromosome. This marker, which was not tested in our dataset, could characterize some M925* samples. Stars highlighted by a grey shading refer to the ancient samples: (1) [41]; (2) [5]; (3) [52]; (4) [38]; their ages, when available, are reported in italics. Dates reported below branches refer to Bayesian estimates of node ages. The networks of the available STR haplotypes (Additional file 8: Table S6) associated with the Q-Y12421 and Q-CTS748 clades and their estimated ages are also illustrated. (PDF 1399 kb)

Additional file 13: **Figure S7.** Phylogeography of the Native American Hgs Q-Z5906 and Q-Z5908 (panels a and b, respectively). Each panel illustrates the phylogenetic relationships of the markers (in different colours) investigated and their pattern of frequency distribution (complete list of samples in Additional file 8: Table S6). Circles without any number refer to one subject. Larger circles refer to the number of subjects specified. The star in panel (a) highlighted by a grey shading refers to an ancient sample [52], subclassified in this study; its relative dating is also reported in italics. Dates reported below branches refer to Bayesian estimates of node ages. The networks of the available STR haplotypes (Additional file 8: Table S6) associated with the Q-Z5906 and Q-Z5908 clades and their estimated ages are also illustrated. (PDF 1080 kb)

Additional file 14: **Figure S8.** Phylogeography of the Native American branch Q-Z780 and its sub-branches. The panel illustrates the phylogenetic relationships of the markers (in different colours) investigated and their pattern of frequency distribution (complete list of samples in Additional file 8: Table S6). The phylogenetic position of SA29 [25], not assessed in our samples, is inferred and indicated with a red dashed line. Circles without any number refer to one subject. Larger circles refer to the number of subjects specified. Stars highlighted by a grey shading refer to ancient samples: (1) [40], (2) [5], (3) [42]; their ages, when available, are reported in italics. Dates reported below branches refer to Bayesian estimates of node ages. The networks of the available STR haplotypes associated with the Q-Z780 sub-branches and their estimated ages are also illustrated. (PDF 1310 kb)

Additional file 15: **Figure S9.** Female perspective (mtDNA) of the main migratory events between Beringia/Asia and North America. (PDF 342 kb)

Additional file 16: **Figure S10.** Comparison of the 51 ancient samples carrying informative Y-chromosomes haplogroups [4, 5, 28, 33, 38, 40–42, 52]. (PDF 115 kb)

Additional file 17: Commands used for processing Y-chromosome WGS. (DOCX 20 kb)
