# Supplementary figures and images for: Analysis of the human Y-chromosome haplogroup Q characterizes ancient population movements in Eurasia and the Americas
Source: BMC Biol. 2019 Jan 24;17:3. doi: 10.1186/s12915-018-0622-4 (PMC6345020; doi:10.1186/s12915-018-0622-4)

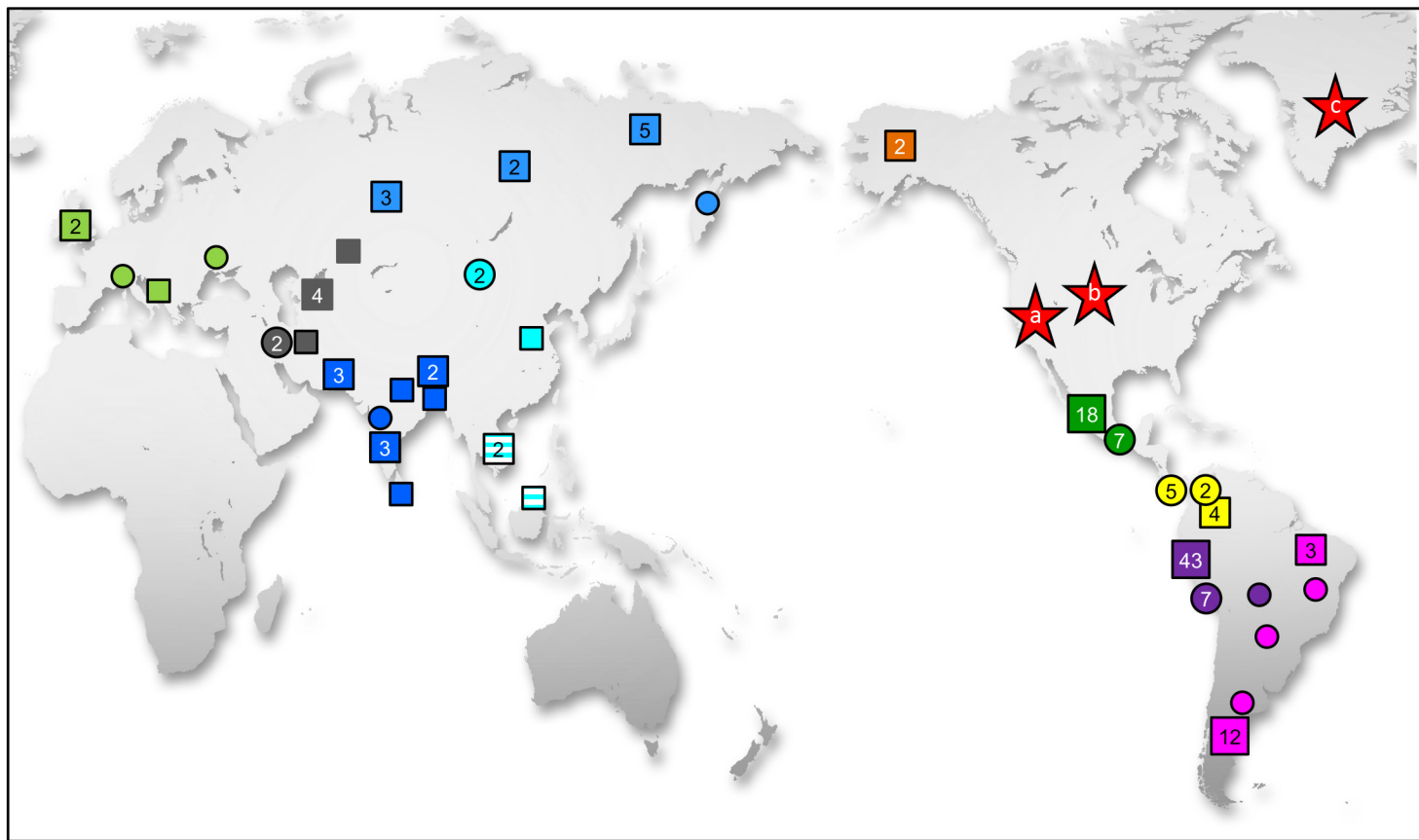

Supplement: Supplementary file 2 — Figure S1. Geographic origin of the subjects included in the phylogenetic analysis and listed in Additional file 1: Table S1. Circles and squares indicate modern samples from this study and from the literature, respectively. Stars indicate ancient samples: a-Kennewick; b-Anzick-1; c-Saqqaq. Colours identify different geographic macro-areas. When more than one subject is from the same area, the number of subjects is reported inside the symbol. (PDF 2653 kb) [file 12915_2018_622_MOESM2_ESM.pdf]

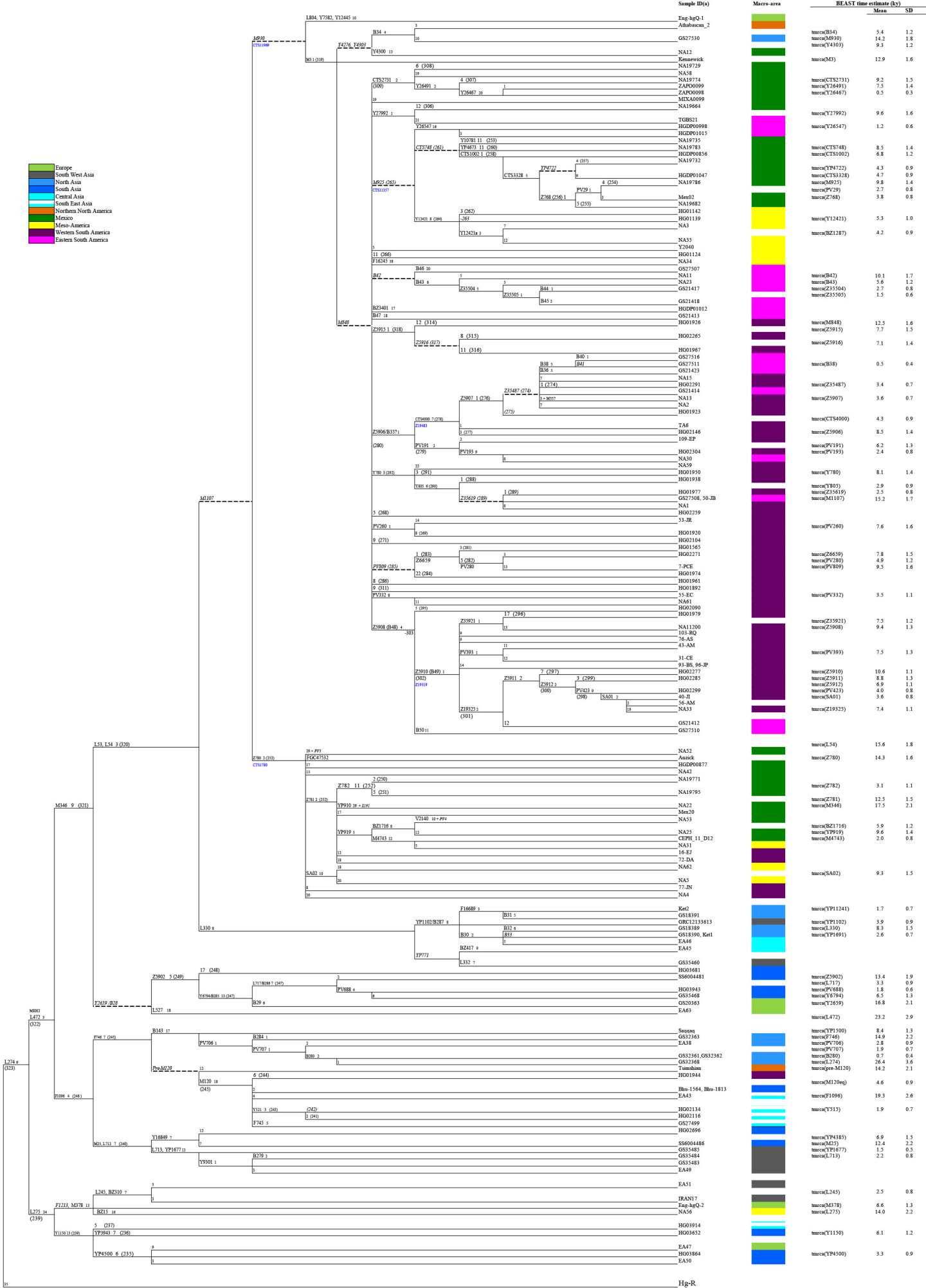

Supplement: Supplementary file 5 — Figure S2. Detailed version of the most parsimonious (MP) tree represented in Fig. 1 and estimated ages of the identified sub-haplogroups. Notes: The length of each branch is not proportional to its age estimate. For each branch, the name of the defining marker(s) and the number of mutations are reported. Markers in italics are outside the sequenced fragments and the relative branches are dotted. Nomenclature in blue colour is according to Jota et al. [25] while names in parentheses are according to Poznik et al. [29]. (a) Sample information in Additional file 1: Table S1. (PDF 962 kb) [file 12915_2018_622_MOESM5_ESM.pdf]

(a) Q-Y2659

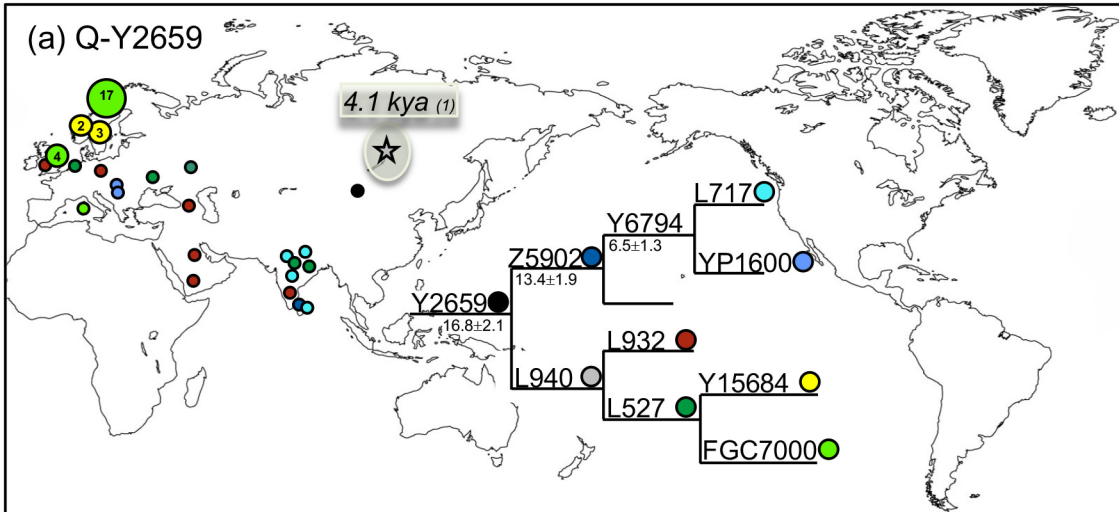

(b) Q-L53

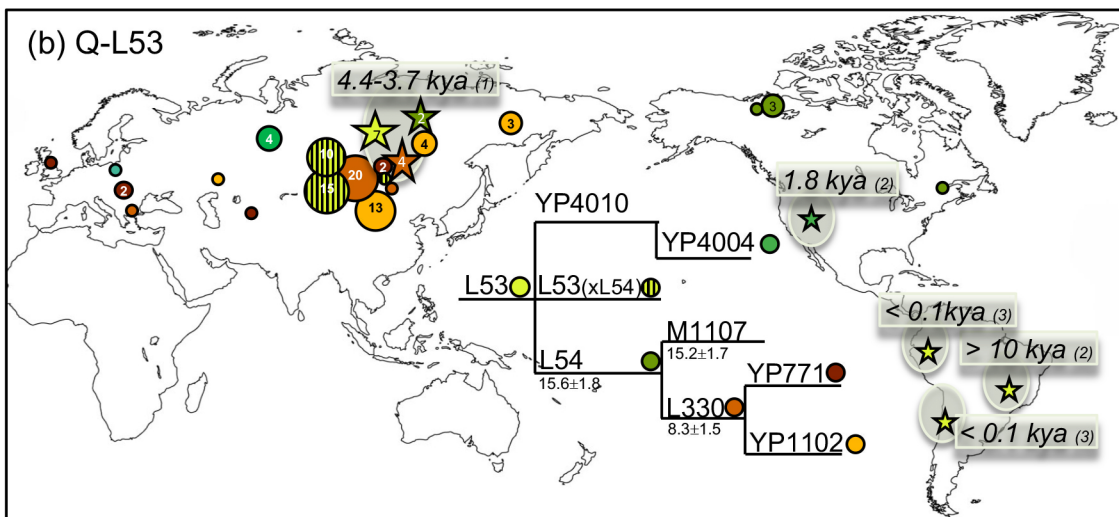

(c) Q-L804

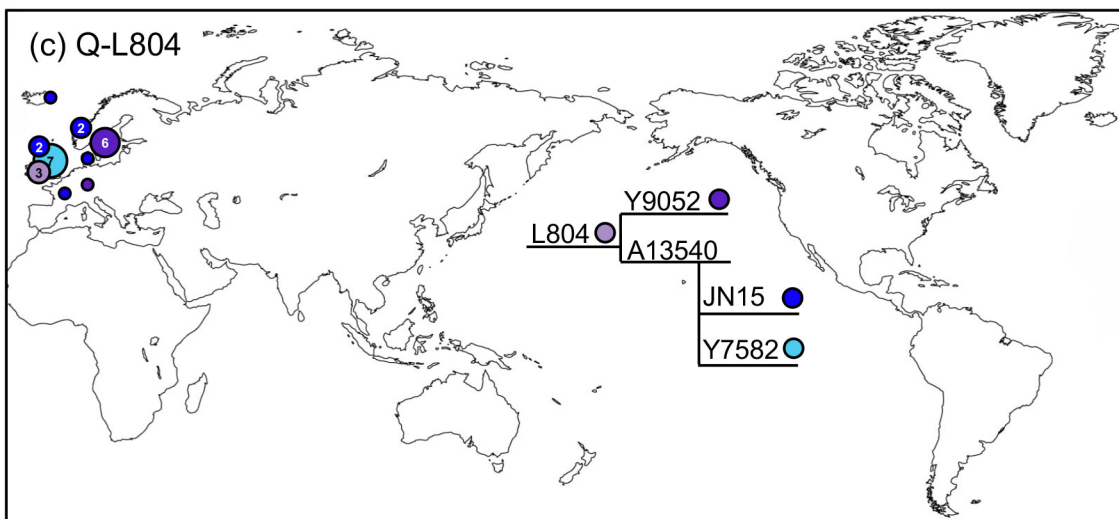

Supplement: Supplementary file 10 — Figure S4. Phylogeography of the branches Q-Y2659, Q-L53 and Q-L804 (panels a, b and c, respectively). Each panel illustrates the phylogenetic relationships of the markers (in different colours) investigated per each branch and their pattern of frequency distribution (complete list of samples in Additional file 8: Table S6). Circles without any number refer to one subject. Larger circles refer to the number of specified subjects. Stars highlighted by a grey shading refer to ancient samples: (1) [75]; (2) [38]; (3) [42]; their relative dating is also reported in italics. Dates reported below branches refer to Bayesian estimates of node ages. (PDF 1183 kb) [file 12915_2018_622_MOESM10_ESM.pdf]

Q-Y4276

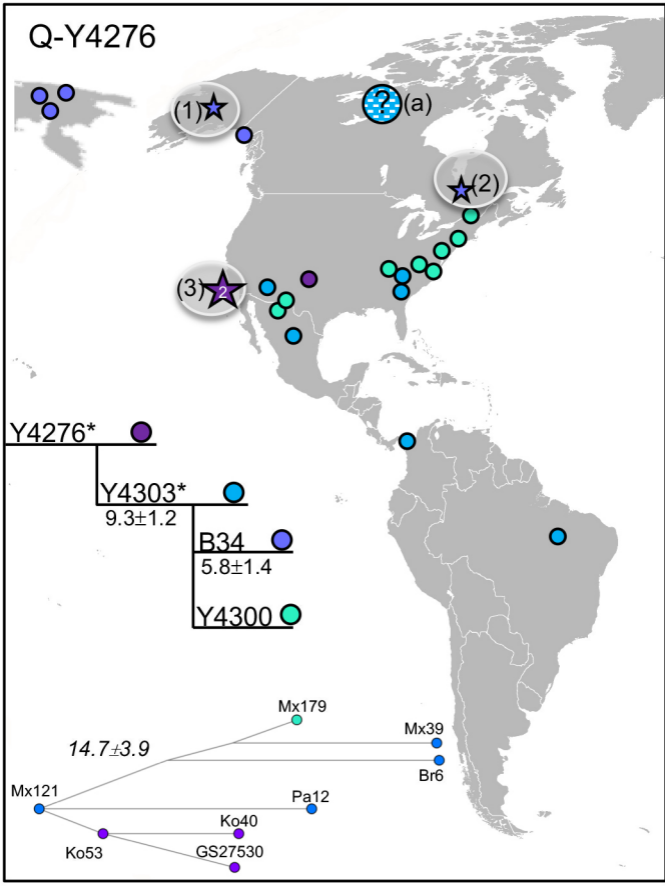

Supplement: Supplementary file 11 — Figure S5. Phylogeography of the Native American branch Q-Y4276. The panel illustrates the phylogenetic relationships of the markers (in different colours) and their pattern of frequency distribution (complete list of samples in Additional file 8: Table S6). Circles without any number refer to one subject. Larger circles refer to the number of specified subjects. Stars highlighted by a grey shading refer to ancient samples: 1) 523a, Alaskan Athabaskan; 2) RM-85, 618–518 ya; 3) SN-11 and SN-38, Late San Nicolas, 1172 ± 39 ya [5]. Dates reported below branches refer to Bayesian estimates of node ages. The network of the available STR haplotypes (Additional file 8: Table S6) and the estimated age associated with this clade are also shown. (a) Q-M3 samples not better sub-classified that could belong, at least in part, to Q-Y4276 [19]. (PDF 312 kb) [file 12915_2018_622_MOESM11_ESM.pdf]

# Q-M925

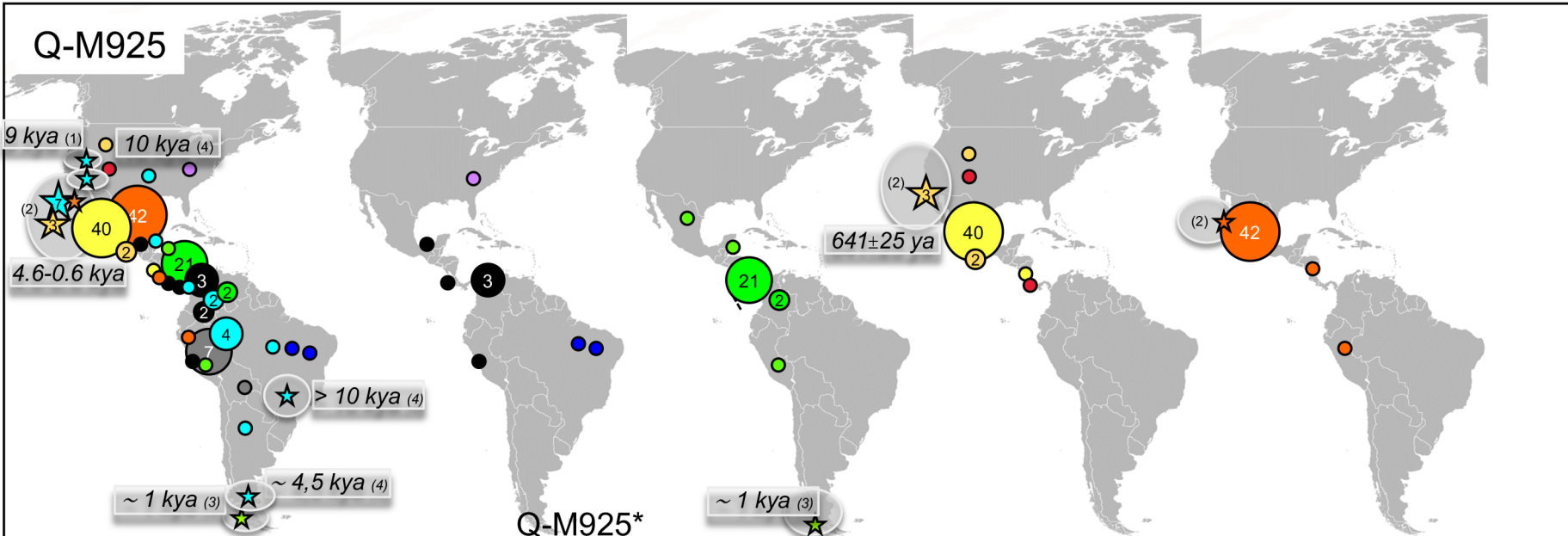

Q-M925<sub>Total</sub>

Q-BZ4012 & Q-Y26547

Q-Y12421

Q-CTS748\*

Q-CTS1002

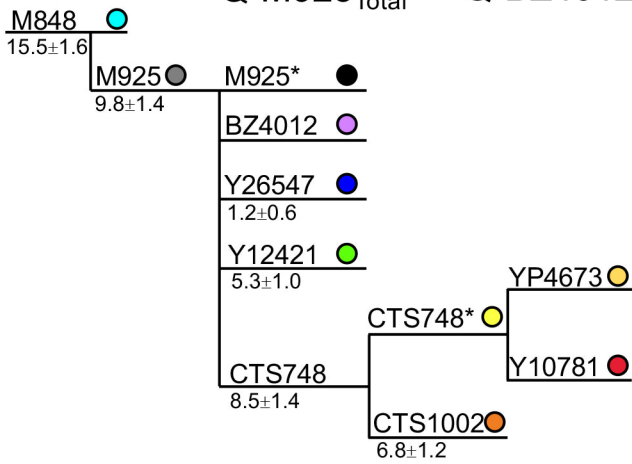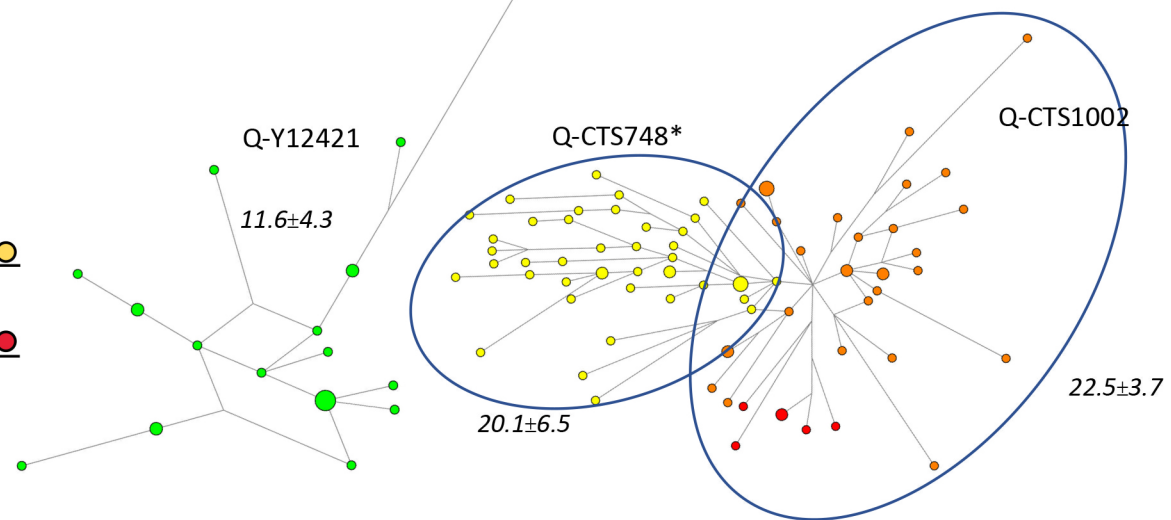

Supplement: Supplementary file 12 — Figure S6. Phylogeography of the Native American branch Q-M925 and its sub-branches. The panel illustrates the phylogenetic relationships of the markers (in different colours) and their pattern of frequency distribution (complete list of samples in Additional file 8: Table S6). Circles without any number refer to one subject. Larger circles refer to the number of specified subjects. Q-BZ4012 has been reported in YFull tree (YTree v6.02 - https://www.yfull.com/tree/Q/) where it is represented by a North Native American Y chromosome. This marker, which was not tested in our dataset, could characterize some M925* samples. Stars highlighted by a grey shading refer to the ancient samples: (1) [41]; (2) [5]; (3) [52]; (4) [38]; their ages, when available, are reported in italics. Dates reported below branches refer to Bayesian estimates of node ages. The networks of the available STR haplotypes (Additional file 8: Table S6) associated with the Q-Y12421 and Q-CTS748 clades and their estimated ages are also illustrated. (PDF 1399 kb) [file 12915_2018_622_MOESM12_ESM.pdf]

(a) Q-Z5906

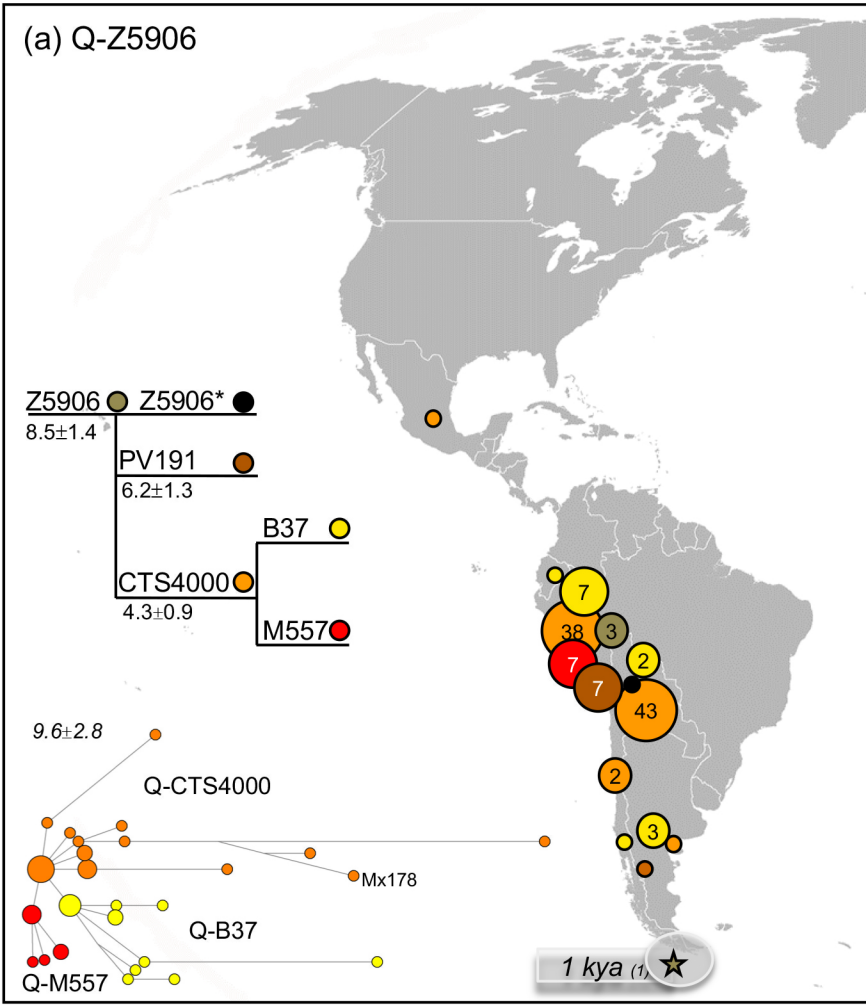

(b) Q-Z5908

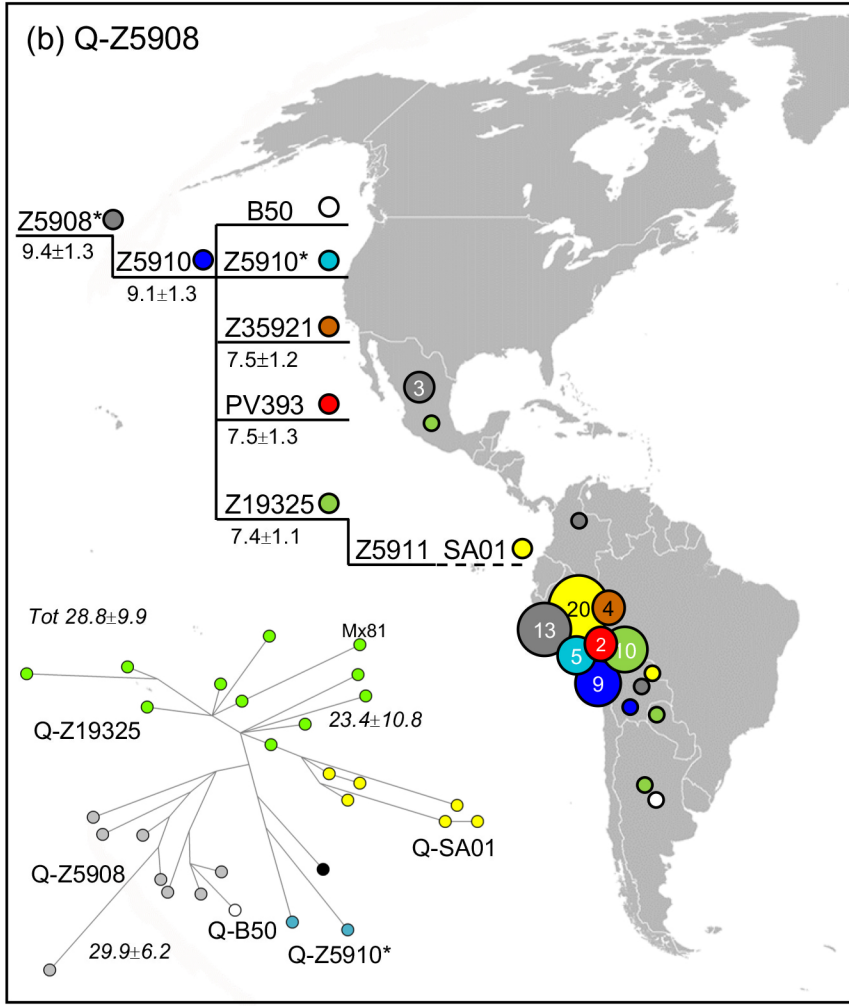

Supplement: Supplementary file 13 — Figure S7. Phylogeography of the Native American Hgs Q-Z5906 and Q-Z5908 (panels a and b, respectively). Each panel illustrates the phylogenetic relationships of the markers (in different colours) investigated and their pattern of frequency distribution (complete list of samples in Additional file 8: Table S6). Circles without any number refer to one subject. Larger circles refer to the number of subjects specified. The star in panel (a) highlighted by a grey shading refers to an ancient sample [52], subclassified in this study; its relative dating is also reported in italics. Dates reported below branches refer to Bayesian estimates of node ages. The networks of the available STR haplotypes (Additional file 8: Table S6) associated with the Q-Z5906 and Q-Z5908 clades and their estimated ages are also illustrated. (PDF 1080 kb) [file 12915_2018_622_MOESM13_ESM.pdf]

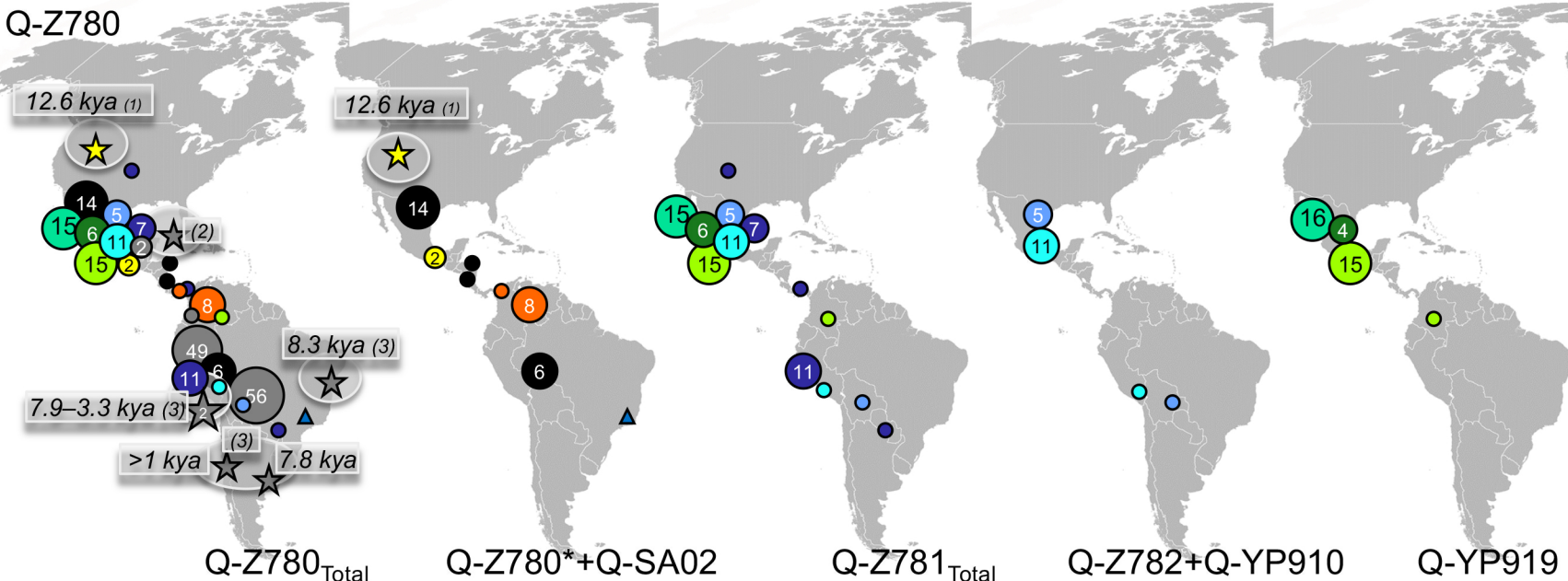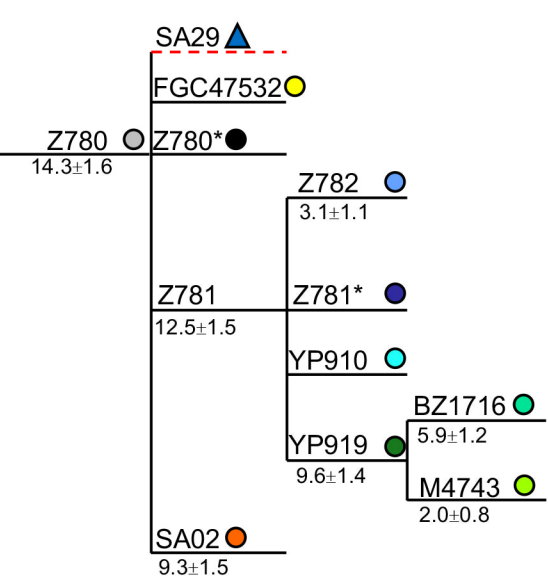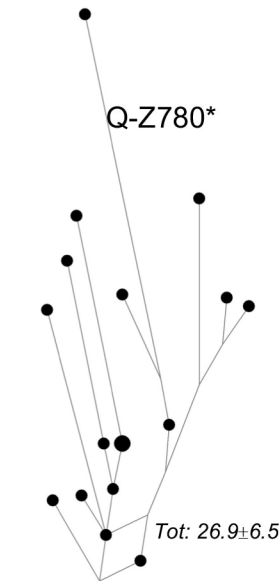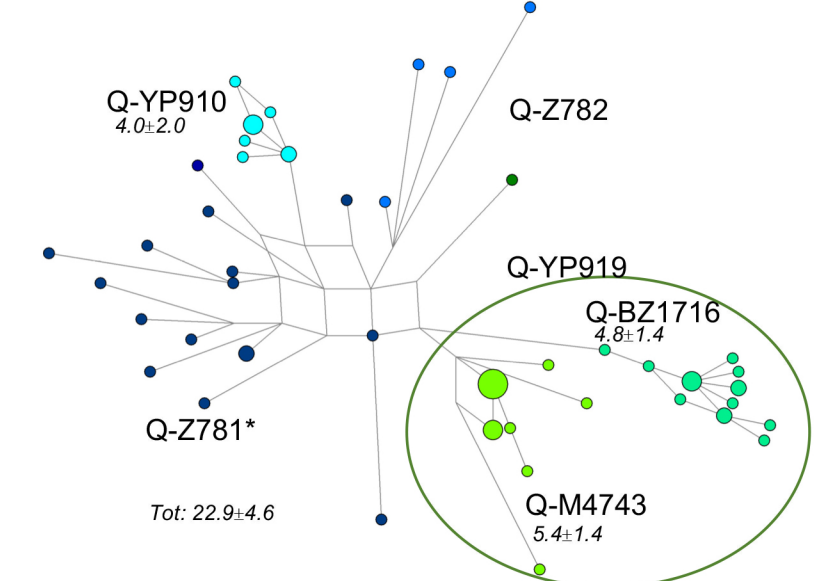

Supplement: Supplementary file 14 — Figure S8. Phylogeography of the Native American branch Q-Z780 and its sub-branches. The panel illustrates the phylogenetic relationships of the markers (in different colours) investigated and their pattern of frequency distribution (complete list of samples in Additional file 8: Table S6). The phylogenetic position of SA29 [25], not assessed in our samples, is inferred and indicated with a red dashed line. Circles without any number refer to one subject. Larger circles refer to the number of subjects specified. Stars highlighted by a grey shading refer to ancient samples: (1) [40], (2) [5], (3) [42]; their ages, when available, are reported in italics. Dates reported below branches refer to Bayesian estimates of node ages. The networks of the available STR haplotypes associated with the Q-Z780 sub-branches and their estimated ages are also illustrated. (PDF 1310 kb) [file 12915_2018_622_MOESM14_ESM.pdf]
