# Supplementary material for: Analysis of the human Y-chromosome haplogroup Q characterizes ancient population movements in Eurasia and the Americas
Source: BMC Biol. 2019 Jan 24;17:3. doi: 10.1186/s12915-018-0622-4 (PMC6345020; doi:10.1186/s12915-018-0622-4)
Supplement: Supplementary file 15 — Figure S9. Female perspective (mtDNA) of the main migratory events between Beringia/Asia and North America. (PDF 342 kb) [file 12915_2018_622_MOESM15_ESM.pdf]

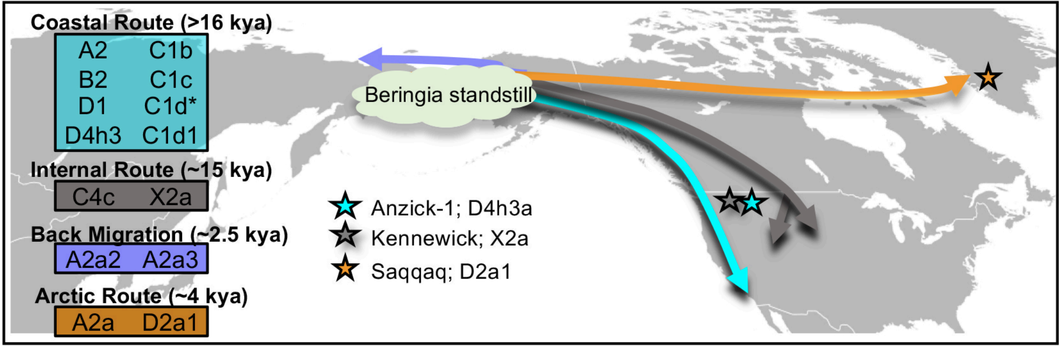
Coastal Route (>16 kya)
A2 C1b
B2 C1c
D1 C1d*
D4h3 C1d1
Internal Route (~15 kya)
C4c X2a
Back Migration (~2.5 kya)
A2a2 A2a3
Arctic Route (~4 kya)
A2a D2a1
Beringia standstill
Anzick-1; D4h3a
Kennewick; X2a
Saqqaq; D2a1
