# Supplementary material for: Analysis of the human Y-chromosome haplogroup Q characterizes ancient population movements in Eurasia and the Americas
Source: BMC Biol. 2019 Jan 24;17:3. doi: 10.1186/s12915-018-0622-4 (PMC6345020; doi:10.1186/s12915-018-0622-4)
Supplement: Supplementary file 16 — Figure S10. Comparison of the 51 ancient samples carrying informative Y-chromosomes haplogroups [4, 5, 28, 33, 38, 40–42, 52]. (PDF 115 kb) [file 12915_2018_622_MOESM16_ESM.pdf]

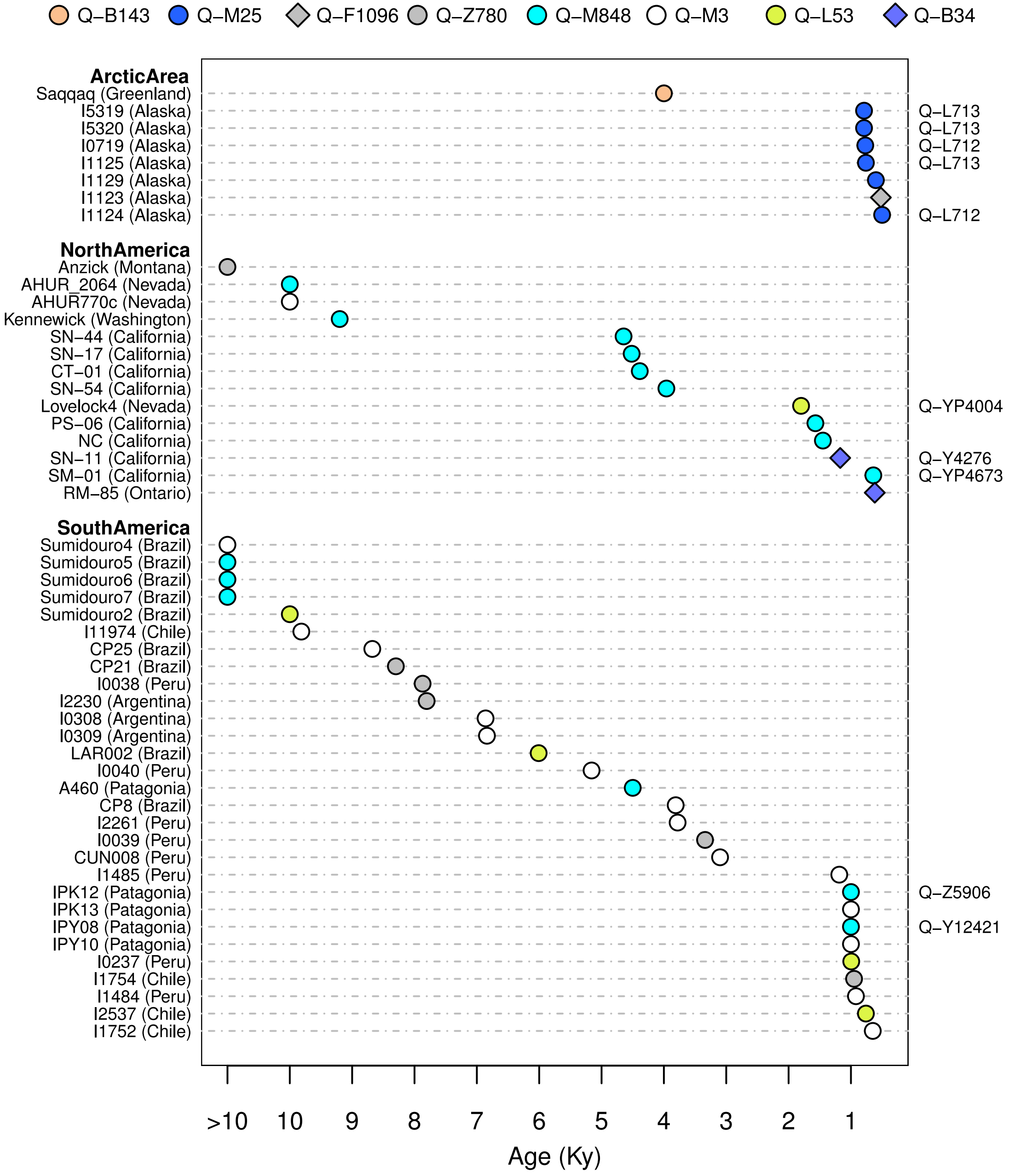

Q−B143
Q−M25
Q−F1096
Q−Z780
Q−M848
Q−M3
Q−L53
Q−B34
ArcticArea
Saqqaq (Greenland)
I5319 (Alaska)
I5320 (Alaska)
I0719 (Alaska)
I1125 (Alaska)
I1129 (Alaska)
I1123 (Alaska)
I1124 (Alaska)
NorthAmerica
Anzick (Montana)
AHUR_2064 (Nevada)
AHUR770c (Nevada)
Kennewick (Washington)
SN−44 (California)
SN−17 (California)
CT−01 (California)
SN−54 (California)
Lovelock4 (Nevada)
PS−06 (California)
NC (California)
SN−11 (California)
SM−01 (California)
RM−85 (Ontario)
SouthAmerica
Sumidouro4 (Brazil)
Sumidouro5 (Brazil)
Sumidouro6 (Brazil)
Sumidouro7 (Brazil)
Sumidouro2 (Brazil)
I11974 (Chile)
CP25 (Brazil)
CP21 (Brazil)
I0038 (Peru)
I2230 (Argentina)
I0308 (Argentina)
I0309 (Argentina)
LAR002 (Brazil)
I0040 (Peru)
A460 (Patagonia)
CP8 (Brazil)
I2261 (Peru)
I0039 (Peru)
CUN008 (Peru)
I1485 (Peru)
IPK12 (Patagonia)
IPK13 (Patagonia)
IPY08 (Patagonia)
IPY10 (Patagonia)
I0237 (Peru)
I1754 (Chile)
I1484 (Peru)
I2537 (Chile)
I1752 (Chile)
Q−L713
Q−L713
Q−L712
Q−L713
Q−L712
Q−YP4004
Q−Y4276
Q−YP4673
Q−Z5906
Q−Y12421
>10
10
9
8
7
6
5
4
3
2
1
Age (Ky)
