# Supplementary material for: Analysis of the human Y-chromosome haplogroup Q characterizes ancient population movements in Eurasia and the Americas
Source: BMC Biol. 2019 Jan 24;17:3. doi: 10.1186/s12915-018-0622-4 (PMC6345020; doi:10.1186/s12915-018-0622-4)
Supplement: Supplementary file 17 — Commands used for processing Y-chromosome WGS. (DOCX 20 kb) [file 12915_2018_622_MOESM17_ESM.docx]

**Supplementary Information 1**

**Pipeline used to analyse NGS data of the Y chromosome**

BAM files provided by BGI-Tech were visual inspected using Integrative Genomics Viewer (IGV, [[1]](https://paperpile.com/c/62rWDk/WF1o)).
SAMtools [[2]](https://paperpile.com/c/62rWDk/HndL) and BCFtools were used for manipulating alignments including indexing, sorting, merging and generating alignments in a per-position format (VCF File).

**Novel Variants calling and Identification of Structural Variants**

In order to accelerate the analyses, the sequences were shortened to the region of our
interest with the command

samtools view -bh initial.bam chrY: 2,689,001-19,550,033 > filtered.bam

in which the initial.bam file was the original file given by BGI-Tech and the region of our interest is the one following the two dots.

Then variant calling was performed with the following command:

samtools mpileup –g –f chrY.fa filtered.bam > output.bcf

In which the reference sequence used was AC_000156.

BCF files were trimmed for the 5,274 fragments of Scozzari and colleagues [[3]](https://paperpile.com/c/62rWDk/8J99) selected within our region of interest with the command:

bcftools view –I –l positions.bed outputfile.bcf > variantsfile.vcf

Positions.bed file contains the coordinates of all the fragments in Scozzari [[3]](https://paperpile.com/c/62rWDk/8J99).

In order to ascertain the depth of a candidate mutation the following command was run

samtools depth -q0 -Q0 -b positions.bed inputfile.bam > outputfile.depth

in which the positions.bed is the same file cited above.

Finally two main criteria were then used to confirm the validity of a candidate mutation: the quality score of consensus (QS) present in the VCF file provided by the BGI and the difference between the depth and the total number of reads for the two best bases.

Variant calls with QS = 99, depth ≥ 4 and difference < 1 were considered true mutations. Variant calls with QS ≤ 90 or QS > 90 but difference > 4 were indicated as Not Available (NA) to discard potential false SNP calls, while calls with 90 ≤ QS ≤99 and 1≤difference≤4 were additionally inspected by visual examination of .bam files through IGV.

**Extracting novel variants from published data**

Y chromosomes belonging to haplogroups A00, R1b and Q were gathered from previous publications [[3–10]](https://paperpile.com/c/62rWDk/8J99+4AKP+W3sF+8QeX+iNkP+EkHo+FJ7u+cGcD).

The samples available as Complete Genomics Master Variation format or as BAM file were converted to VCF format files using the C compiled tool masterVar2VCFv41_rev1.c and samtools, the latter as described above.

For data already available as VCF, the variants not included in the region of interest were discarded using tabix [[11]](https://paperpile.com/c/62rWDk/lRLI) with the command:

tabix -fh input.vcf positions.bed > output_total.vcf

In which the position.bed is the same file of above.

**Extracting variants from ancient data**

Ancient Y-chromosome VCF and BAM files [[12–14]](https://paperpile.com/c/62rWDk/bZwY+xufy+R2hm) belonging to haplogroup Q sub-lineages were visually inspected with both IGV and in-house scripts for only the novel variants previously extracted from modern data. Variants that did not match the previous haplogroup classification [[8, 15]](https://paperpile.com/c/62rWDk/EkHo+JslI) were discarded.

**References**

[1. Thorvaldsdóttir H, Robinson JT, Mesirov JP. Integrative Genomics Viewer (IGV): high-performance genomics data visualization and exploration. Brief Bioinform. 2013;14:178–92.](http://paperpile.com/b/62rWDk/WF1o)

[2. Li H, Handsaker B, Wysoker A, Fennell T, Ruan J, Homer N, et al. The Sequence Alignment/Map format and SAMtools. Bioinformatics. 2009;25:2078–9.](http://paperpile.com/b/62rWDk/HndL)

[3. Scozzari R, Massaia A, Trombetta B, Bellusci G, Myres NM, Novelletto A, et al. An unbiased resource of novel SNP markers provides a new chronology for the human Y chromosome and reveals a deep phylogenetic structure in Africa. Genome Res. 2014;24:535–44.](http://paperpile.com/b/62rWDk/8J99)

[4. Raghavan M, Skoglund P, Graf KE, Metspalu M, Albrechtsen A, Moltke I, et al. Upper Palaeolithic Siberian genome reveals dual ancestry of Native Americans. Nature. 2014;505:87–91.](http://paperpile.com/b/62rWDk/4AKP)

[5. Hallast P, Jobling MA. The Y chromosomes of the great apes. Hum Genet. 2017;136:511–28.](http://paperpile.com/b/62rWDk/W3sF)

[6. Zhou D, Udpa N, Ronen R, Stobdan T, Liang J, Appenzeller O, et al. Whole-genome sequencing uncovers the genetic basis of chronic mountain sickness in Andean highlanders. Am J Hum Genet. 2013;93:452–62.](http://paperpile.com/b/62rWDk/8QeX)

[7. Trombetta B, D’Atanasio E, Massaia A, Ippoliti M, Coppa A, Candilio F, et al. Phylogeographic refinement and large scale genotyping of human Y chromosome haplogroup E provide new insights into the dispersal of early pastoralists in the African continent. Genome Biol Evol. 2015;7:1940–50.](http://paperpile.com/b/62rWDk/iNkP)

[8. Karmin M, Saag L, Vicente M, Wilson Sayres MA, Järve M, Talas UG, et al. A recent bottleneck of Y chromosome diversity coincides with a global change in culture. Genome Res. 2015;25:459–66.](http://paperpile.com/b/62rWDk/EkHo)

[9. Underhill PA, Poznik GD, Rootsi S, Järve M, Lin AA, Wang J, et al. The phylogenetic and geographic structure of Y-chromosome haplogroup R1a. Eur J Hum Genet. 2015;23:124–31.](http://paperpile.com/b/62rWDk/FJ7u)

[10. 1000 Genomes Project Consortium, Auton A, Brooks LD, Durbin RM, Garrison EP, Kang HM, et al. A global reference for human genetic variation. Nature. 2015;526:68–74.](http://paperpile.com/b/62rWDk/cGcD)

[11. Li H. Tabix: fast retrieval of sequence features from generic TAB-delimited files. Bioinformatics. 2011;27:718–9.](http://paperpile.com/b/62rWDk/lRLI)

[12. Rasmussen M, Li Y, Lindgreen S, Pedersen JS, Albrechtsen A, Moltke I, et al. Ancient human genome sequence of an extinct Palaeo-Eskimo. Nature. 2010;463:757–62.](http://paperpile.com/b/62rWDk/bZwY)

[13. Rasmussen M, Anzick SL, Waters MR, Skoglund P, DeGiorgio M, Stafford TW Jr, et al. The genome of a Late Pleistocene human from a Clovis burial site in western Montana. Nature. 2014;506:225–9.](http://paperpile.com/b/62rWDk/xufy)

[14. Rasmussen M, Sikora M, Albrechtsen A, Korneliussen TS, Moreno-Mayar JV, Poznik GD, et al. The ancestry and affiliations of Kennewick Man. Nature. 2015;523:455–8.](http://paperpile.com/b/62rWDk/R2hm)

[15. Scheib CL, Li H, Desai T, Link V, Kendall C, Dewar G, et al. Ancient human parallel lineages within North America contributed to a coastal expansion. Science. 2018;360:1024–7.](http://paperpile.com/b/62rWDk/JslI)
