# Supplementary material for: Analysis of the human Y-chromosome haplogroup Q characterizes ancient population movements in Eurasia and the Americas
Source: BMC Biol. 2019 Jan 24;17:3. doi: 10.1186/s12915-018-0622-4 (PMC6345020; doi:10.1186/s12915-018-0622-4)
Supplement: Supplementary file 9 — Figure S3. Phylogeography of the branches Q-Y1150, Q-M378 and Q-F1096 (panels a, b and c, respectively). Each panel illustrates the phylogenetic relationships of the markers (in different colours) investigated per each branch and their pattern of frequency distribution (complete list of samples in Additional file 8: Table S6). Circles without any number refer to one subject. Larger circles refer to the number of specified subjects. Stars highlighted by a grey shading refer to ancient samples: (1) [74], (2) [75], (3) [76], (4) [28]; their relative dating, when available, is also reported in italics. Dates reported below branches refer to Bayesian estimates of node ages. (PDF 1420 kb) [file 12915_2018_622_MOESM9_ESM.pdf]

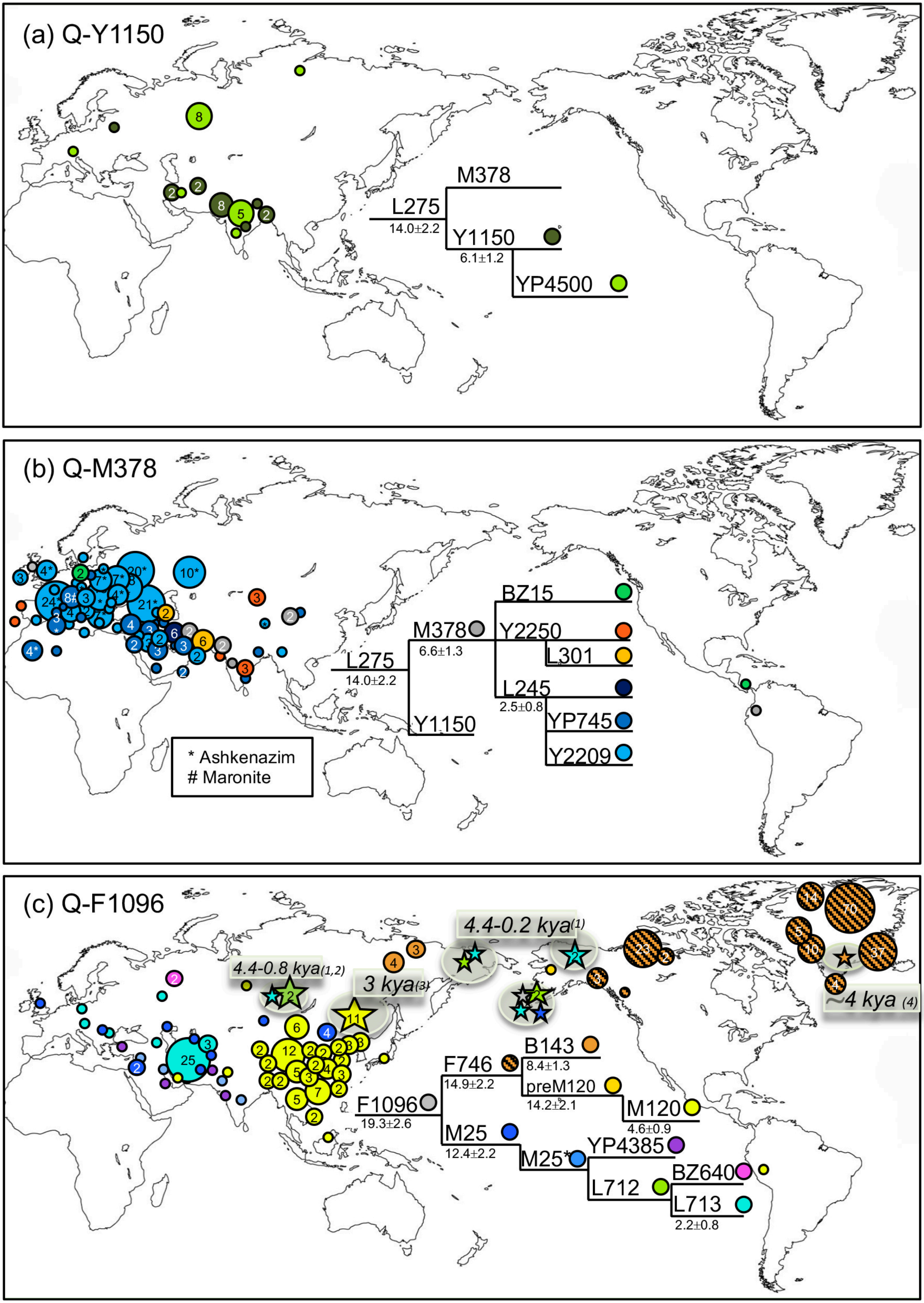

(a) Q-Y1150
M378
L275
14.0±2.2
Y1150
6.1±1.2
YP4500
(b) Q-M378
BZ15
M378
6.6±1.3
Y2250
L301
L275
14.0±2.2
L245
2.5±0.8
YP745
Y1150
Y2209
* Ashkenazim
# Maronite
(c) Q-F1096
4.4-0.2 kya(1)
4.4-0.8 kya(1,2)
3 kya(3)
~4 kya (4)
B143
8.4±1.3
F746
14.9±2.2
preM120
14.2±2.1
M120
4.6±0.9
F1096
19.3±2.6
M25
12.4±2.2
M25*
YP4385
BZ640
L712
L713
2.2±0.8
